# Unequal treatment toward copartisans versus non-copartisans is reduced when partisanship can be falsified

**DOI:** 10.1371/journal.pone.0244651

**Published:** 2021-01-27

**Authors:** Maria Abascal, Kinga Makovi, Anahit Sargsyan

**Affiliations:** 1 Department of Sociology, New York University, New York, New York, United States of America; 2 Social Science Division, New York University Abu Dhabi, Abu Dhabi, UAE; Universidad Loyola Andalucia Cordoba, SPAIN

## Abstract

Studies show that Democrats and Republicans treat copartisans better than they do non-copartisans. However, party affiliation is different from other identities associated with unequal treatment. Compared to race or gender, people can more easily falsify, i.e., lie about, their party affiliation. We use a behavioral experiment to study how people allocate resources to copartisan and non-copartisan partners when partners are allowed to falsify their affiliation and may have incentives to do so. When affiliation can be falsified, the gap between contributions to signaled copartisans and signaled non-copartisans is eliminated. This happens in part because some participants—especially strong partisans—suspect that partners who signal a copartisan affiliation are, in fact, non-copartisans. Suspected non-copartisans earn less than both partners who signal that they are non-copartisans and partners who withhold their affiliation. The findings reveal an unexpected upside to the availability of falsification: at the aggregate level, it reduces unequal treatment across groups. At the individual-level, however, falsification is risky.

## Introduction

People are generally more prosocial toward those with whom they share a group identity, whether that identity is based on race or ethnicity [1–4], nationality [5], organizational membership [6, 7], or some other trait, like religion [8, 9]. Even identities induced by researchers are associated with unequal treatment across groups [10–12]. Explanations for unequal treatment include exclusionary preferences (e.g., [13]) and strategic expectations [14–16].

Not all identities are, like race or gender, readily observable; some can be hard to discern from how someone looks or speaks. Against this backdrop, individuals may attempt to avoid being mistreated by strategically misrepresenting their identities. This is especially true when resources—whether economic, political, or social—induce strategic incentives to present oneself in a certain way. In workplaces, for example, people may regularly face both dissimilar others and pressure to manage impressions. Misrepresentation can take two forms: (a) falsification, i.e., expressing an opinion or identity in public that differs from the one held privately [17, 18], or (b) non-disclosure, i.e., refraining from expressing one’s opinion or identity in public [19–21].

People do not invariably misrepresent when given an opportunity and incentive to do so. Even when dishonesty may be profitable, some people are unconditionally honest, whereas others are unconditionally dishonest [22, 23]. For many others, honesty is malleable and sensitive to situational features, most notably, monitoring [22, 24]. When discovered, dishonesty can incur material and reputational sanctions [25]. However, even absent the possibility of discovery or sanctioning, evidence suggests a strong, psychological disposition to tell the truth [18, 22, 26], though the evidence for widespread “lying aversion” has recently come under scrutiny [27].

Psychological dispositions notwithstanding, some people lie. This simple fact introduces uncertainty into interactions in which one or more parties have both an ability and an incentive to misrepresent who they are. How do people treat those about whose opinions or identities they are uncertain? Specifically, what happens to the aggregate allocation of resources to people who signal an ingroup versus an outgroup identity when people are given the ability to falsify their identities and an incentive to do so? And, what cost does someone incur when she is suspected of falsifying her identity? Is she treated as she would have been had she revealed an outgroup identity, or does she incur an additional cost for possibly lying? These questions, which have not yet been considered by prior research, are the subject of the present study.

In the S1 File we formally derive the ways in which behavior might be affected by uncertainty stemming from the availability of falsification. With plausible assumptions—specifically, that alters who signal that they are outgroup members are not believed to be ingroup members—the effect of uncertainty on unequal treatment hinges on four parameters: (1) the propensity to believe an alter who signals an ingroup affiliation, (2) the reward for signaling an ingroup affiliation when that affiliation is believed, (3) the punishment for signaling an ingroup affiliation when that affiliation is not believed, and (4) the reward for signaling an outgroup affiliation. (Signaling an outgroup affiliation may be punished rather than rewarded if the choice to do so is seen as insolent when falsification is available.) In brief, uncertainty may exacerbate unequal treatment between those who signal an ingroup or outgroup affiliation, it may mitigate it, or it may leave this gap unchanged.

We explore these questions for the case of political partisanship in the United States. We focus on political partisanship for two reasons. First, partisanship is an increasingly divisive and salient social identity in the United States [28–31] as well as other countries [32]. Both Democrats and Republicans behave more prosocially toward copartisans than non-copartisans [33–35] but see [36], and they are willing to incur costs to express preferences for copartisans [37]. In fact, Americans are more willing to discriminate openly against non-copartisans than racial outgroup members [30, 38]. This might be due to increasingly strong norms against discrimination on the basis of ascribed identities, like race or gender. This thesis comes with two caveats, though: (1) gender and, by some accounts, race are becoming increasingly fluid (an issue to which we return), and (2) identities that are socially understood to be unchangeable have been the basis of singular violence, precisely because they were understood to be unchangeable [39]. Second, in contrast with identities that are more immediately observable—most notably race (see [40] on the “ocularity” of race)—partisanship can be readily concealed or falsified, especially to weak ties as opposed to strong ones. For example, selective non-disclosure may be prevalent in workplaces, where people face strong incentives to manage impressions [21] in interactions with politically dissimilar others [41].

To anticipate our findings, when partners are neither able to falsify nor withhold their affiliation, participants are more generous to partners who signal that they are copartisans than to partners who signal that they are non-copartisans. However, when partners are able to falsify their affiliation, participants are equally generous, in aggregate, to partners who signal that they are copartisans and to partners who signal that they are non-copartisans. This happens largely because participants—and especially strong partisans—suspect that some partners who signal a copartisan affiliation are, in fact, non-copartisans. These suspected non-copartisans receive the least generous contributions. In sum, the possibility for falsification makes it risky to signal agreement; one has more to lose from signaling agreement and not being believed than from signaling disagreement (accurate or not).

Why do partners who are suspected of dishonesty incur a penalty, above and beyond the one associated with identifying with an outgroup? Is it because they are suspected of lying about themselves in order to elicit an undeserved gain? Alternatively, or in addition, are they punished because, in not presenting their “true” identity, they are failing to reciprocate the disclosure of the participant’s identity, thereby signaling mistrust in the participant and, by possible extension, the participant’s ingroup? To adjudicate between these mechanisms, we examine behavior a setting in which partners can conceal (but not falsify) their identity. The comparison yields support for the first pathway: participants are less generous to partners they suspect of dishonesty than to partners who withhold their affiliation. In sum, suspected dishonesty is punished above and beyond a failure to reciprocate an information exchange.

## Experimental game

We designed and fielded an incentivized survey experiment with US adult participants, recruited through Amazon Mechanical Turk (MTurk). At the beginning of the survey, participants reported their party identification in two steps. First, participants reported whether they identified as a Republican, Democrat, Independent, or “Something else.” Prospective participants who selected “Independent” or “Something else” were screened out. Those who selected “Republican” or “Democrat” were asked to report the strength of this identification, hereafter “party-strength,” on a six-point scale ranging from “Strong Republican” to “Strong Democrat.” For more details on our recruitment strategy and screening criteria, see Materials and methods; for the survey instrument, see the S1 File.

Next, participants played a simple Dictator Game (DG), which is used to measure prosocial behavior [42, 43]. All participants were assigned to the role of “dictator,” or “Player A.” In this role, they were asked to split $2.00 between themselves and a partner, or “Player B.” Players B were other MTurk workers. Their responses—which we collected separately, as part of the same experiment—are not analyzed here.

The motivations for prosocial behavior are both wide-ranging and the subject of an active area of research. They may include fear of punishment, impression and reputation management, norm compliance, and altruism (which has evolved to be parochial in nature), among others. In this study, we assume that these motivations do not differ dramatically across the experimental conditions described next, and therefore, that observed differences between contributions to copartisans and non-copartisans across conditions are due to the experimental manipulations.

Participants were assigned to one of three conditions: **baseline**, **falsification**, or **non-disclosure**. Assignment was random within each of the six party-strengths. Across all conditions, Players A were informed that (1) Player B answered the same two questions about their party identification and (2) Player B then saw Player A’s party-strength.

In the **baseline** condition, Player A was further informed that they would learn Player B’s party-strength and then they would decide how to split the $2.00. In fact, participants were assigned to learn one of the six possible party-strengths for Player B (e.g., “Strong Republican”), producing six sub-conditions.

In the **falsification** condition, Player A was also informed that they would learn Player B’s party-strength before deciding how to split the $2.00. In addition, they learned that Player B was given the option to change their answer to the party-strength question after learning Player A’s answer to this same question. Like the baseline condition, the falsification condition comprises six sub-conditions corresponding to the six party-strengths available to Players B.

In the **non-disclosure** condition, Player A was also informed that they would learn Player B’s party-strength before deciding how to split the $2.00. In addition, they learned that Player B was given the option to withhold their party-strength after learning Player A’s. The non-disclosure condition thus comprises seven sub-conditions to which participants were assigned: six possible party-strengths for Player B and a non-disclosure response. In sum, participants within each self-declared party-strength were assigned to one of 19 sub-conditions, determined by experimental condition and Player B’s party-strength.

After splitting the $2.00, participants were asked to describe in a few sentences how they made their decision. We analyze these qualitative responses to gain insight into the mechanisms underlying differences in contributions. The S1 File describe the coding of qualitative responses. As a manipulation check, we asked participants to recall their partner’s reported party-strength. Next, we asked participants to report how they believed their partner really identified. A flow-chart of the experimental procedures is presented in S1 Fig in S1 File.

The following analyses are based on those 2,538 participants who met the screening criteria, who correctly answered comprehension-check questions prior to playing the DG, and who successfully took up treatment (more details below and in Materials and Methods, as well as the S1 File, showing that the treatment take-up exclusion does not substantially affect the results). In the main analyses, we aggregate the responses of Democrats and Republicans, and we consider both their DG contributions and their beliefs about their partners. In the S1 File, we disaggregate the analyses for Democrats and Republicans. Briefly, we find that Democrats and Republicans behave similarly toward copartisans and non-copartisans, hence our decision to report pooled results.

MTurk is a popular platform for experimental research [44, 45], including research on political ideology [46]. However, MTurk workers are not representative of the US adult population [47]. S4 Table in S1 File reports demographic characteristics for our analytic sample by experimental condition. Both Democrats and Republicans in our sample are younger and more educated than the average American; the Republicans in our sample are also more likely to be women and have a slightly higher income, while Democrats are more likely to identify as White [48] (S5 and S6 Tables in S1 File).

Our sample contains different numbers of participants who identify with each party-strength, largely as the result of the under-representation of Republicans on MTurk. We therefore weight our observations using two strategies. In the main text, we give equal weight to each party-strength category, ensuring that the results of the aggregate analyses do not depend on the relative numbers of participants in each sub-condition. In the S1 File we also report the main analyses, instead weighting observations so that the sample resembles a nationally representative sample of US Democrats and Republicans, respectively, in terms of observed sociodemographics [49]. For details, see Materials and methods. Results are substantively similar, and differences are noted in the S1 File.

## Results

### Patterns of beliefs

In the baseline condition, where partners were not allowed to change or withhold their affiliation after learning the participant’s, a partner’s affiliation should not have been a source of uncertainty. Results confirm this was largely the case (Table 1). Of participants whose partner signaled that they were a copartisan, 93.61% believed they were a copartisan. Of participants whose partner signaled that they were a non-copartisan, 97.78% believed they were a non-copartisan.

**Table 1:** Share of participants who believed their partner’s signaled identity by the identity signaled, across experimental conditions.

	Signaled copartisan	Signaled non-copartisan
**Baseline**	93.61	97.78
**Falsification**	70.35	99.02
**Non-disclosure**	90.84	98.15

In the falsification condition, where partners could change their affiliation after learning the participant’s, participants should have reasoned that some of the partners who signaled they were copartisans were, in fact, non-copartisans. Indeed, of participants whose partner was a signaled copartisan, just 70.35% believed they were a copartisan. This is significantly lower than the 93.61% of participants in the baseline condition who believed a signaled copartisan was a copartisan (*P* < 0.001, one-sided t-test, H_1_: *μ*_1_ < *μ*_2_). By contrast, of participants whose partner was a signaled non-copartisan, 99.02% believed they were a non-copartisan. This is comparable to the 97.78% of participants in the baseline condition who believed a signaled non-copartisan was a non-copartisan (*P* = 0.159, two-sided t-test).

In the non-disclosure condition, where partners could withhold their affiliation after learning the participant’s, participants should have reasoned that non-copartisans would be more likely to withhold their affiliation. Results confirm this was the case (Table 1). Of participants whose partner was a signaled copartisan, 90.84% believed they were a copartisan. Of participants whose partner was a signaled non-copartisan, 98.15% believed they were a non-copartisan. However, of participants whose partner withheld their party affiliation, a majority (86.42%) believed they were a non-copartisan (*P* < 0.001, two-sided z-test, H_0=0.5_).

In the baseline and non-disclosure conditions, participants should have believed the identity signaled by their partner. These participants “took up treatment.” The following analyses are based on the vast majority of participants (95.52%) in the baseline and non-disclosure conditions who believed a signaled copartisan was, in fact, a copartisan or a signaled non-copartisan was, in fact, a non-copartisan. Results are substantively similar without this exclusion (S26 Table in S1 File). We do not exclude any participants in the non-disclosure condition whose partners withheld their affiliation. Nor do we exclude participants in the falsification condition based on their reported beliefs about their partner’s affiliation. In what follows, we analyze contributions to partners mainly by their signaled identities, rather than their initially reported or “real” identities. In the real world, as in prior studies, only signaled identities are observed.

### Baseline and falsification

Participants could contribute any amount between $0.00 and $2.00, down to the $0.01 increment; however, the vast majority contributed $0.00 (34.99%) or $1.00 (41.65%). Accordingly, we analyze equitable contributions which we define as those where participants gave partners at least half ($1.00) of their endowment (only 17 participants gave more than $1.00). The motivations for prosocial behavior are both wide-ranging and the subject of an active area of research. They may include fear of punishment, impression and reputation management, norm compliance, and altruism (which has evolved to be parochial in nature), among others. In this study, we assume that these motivations do not differ dramatically across experimental conditions, and therefore, that observed differences between contributions to copartisans and non-copartisans across conditions are due to the experimental manipulations. The main analyses focus on share of equitable contributions, because contributions on the continuous scale are not normally distributed. S5 Fig in S1 File reports contributions on the continuous scale; results reveal no substantive differences.

**Fig 1:**
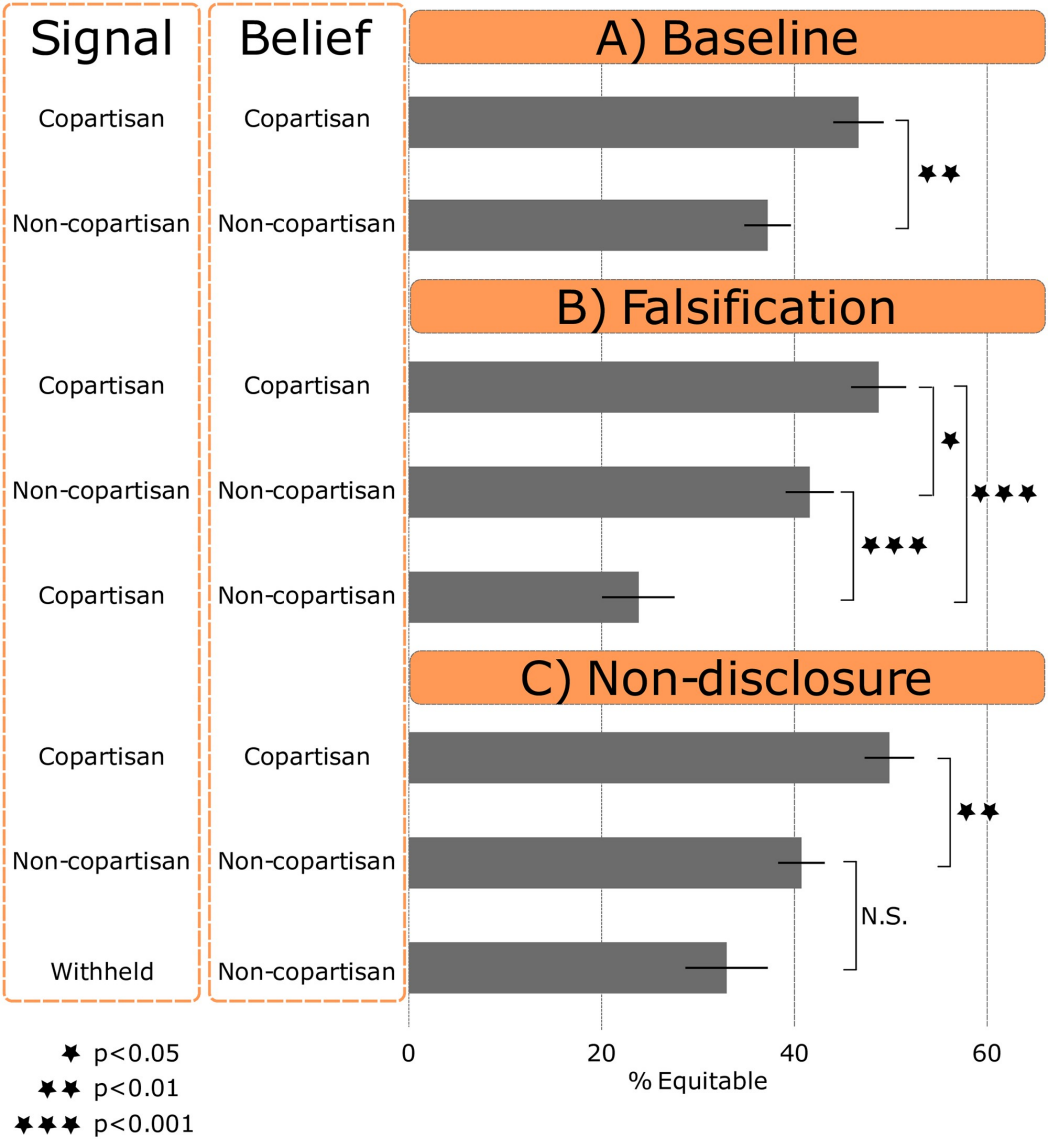
Share of equitable contributions by the identity signaled by partners and the identity believed by participants, across experimental conditions, p-values are based on two-sided t-tests.

Table 2 reports the share of equitable contributions by the signaled identities of partners. Fig 1 reports the share of equitable contributions by the signaled identities of partners, further broken down by the believed identities of partners. For the baseline condition, where signals were reliably believed, Fig 1A reports the same information. Based on previous work, we expect that baseline participants will be more likely to behave equitably toward copartisans than non-copartisans. Indeed, 46.61% of baseline participants with a copartisan partner behaved equitably; by comparison, just 37.34% of baseline participants with a non-copartisan partner behaved equitably (Cohen’s h = 0.19, *P* < 0.01, two-sided t-test).

**Table 2:** Share of equitable contributions by the identity signaled by partners, across experimental conditions.

	Copartisan	Non-copartisan	Withheld
**Baseline**	46.61	37.34	–
**Falsification**	41.78	40.80	–
**Non-disclosure**	50.02	40.24	34.94

Uncertainty in the falsification condition, particularly concerning partners who signal a copartisan affiliation, may reduce the gap in equitable contributions to signaled copartisans and signaled non-copartisans. Indeed, we find that participants in the falsification condition are only slightly less generous to partners who signaled a non-copartisan affiliation than to partners who signaled a copartisan affiliation (Table 2). Specifically, 41.78% of participants behaved equitably toward partners who signaled a copartisan affiliation, compared to 40.80% of participants who behaved equitably toward partners who signaled a non-copartisan affiliation. This small difference, however, is not statistically significant (Cohen’s h = 0.02, *P* = 0.773, two-sided t-test). In sum, the difference between contributions to copartisans and non-copartisans is smaller (it is, in fact, eliminated) in the falsification condition than the baseline condition. See S27 Table in S1 File for results based on a linear probability model controlling for participant demographics confirming this result.

Fig 1B disaggregates equitable contributions in the falsification condition by both the signaled and believed partisanship of partners. The results point to two avenues through which the availability of falsification reduces the gap in contributions to signaled copartisans versus signaled non-copartisans. First, participants make slightly more generous contributions to signaled non-copartisans, as anticipated by work which shows that revealing compromising information can elicit prosocial behavior from others [50]. Specifically, 40.71% of participants behaved equitably toward a non-copartisan whom they believed to be a non-copartisan in the falsification condition. By comparison, 37.34% of participants behaved equitably toward a non-copartisan in the baseline condition. The difference, though, is not statistically significant (*P* = 0.325, two-sided t-test).

Second, participants give signaled copartisans slightly less in the falsification condition than in the baseline condition. 41.78% of participants behaved equitably toward a signaled copartisan in the falsification condition, compared to 46.61% of participants who behaved equitably toward a signaled copartisan in the baseline condition (*P* = 0.087, one-sided t-test, H_1_: *μ*_1_ < *μ*_2_). Aggregating these trends, the shares of participants who behaved equitably toward all partners—copartisan and non-copartisan—were comparable in the falsification condition (41.30%) and the baseline condition 41.77%, (*P* = 0.849, two-sided t-test). In sum, the reduction in the copartisan–non-copartisan gap the does not coincide with an overall reduction in generosity.

Why are participants slightly less generous to signaled copartisans in the falsification condition than the baseline condition? This happens because some participants in the falsification condition believe signaled copartisans are, in fact, non-copartisans. Recall that among participants whose partner was a signaled copartisan, just 70.35% believed they were a copartisan. 49.45% of these participants behaved equitably toward their partners, compared to just 46.61% who behaved equitably toward a signaled copartisan in the baseline condition (though *P* = 0.467, two-sided t-test). By contrast, the participants who believed a signaled copartisan was, in fact, a non-copartisan were substantially less generous. Just 23.92% of these participants behaved equitably toward their partner, significantly less than the 40.72% who behaved equitably toward a signaled non-copartisan in the falsification condition (*P* < 0.001, one-sided t-test, H_1_: *μ*_1_ < *μ*_2_) or the 37.34% who behaved equitably toward a non-copartisan in the baseline condition (*P* < 0.01, one-sided t-test, H_1_: *μ*_1_ < *μ*_2_).

In sum, participants exact a penalty for suspected dishonesty, above and beyond the penalty for being a non-copartisan. Signaling a copartisan identity is therefore risky: a partner who is not believed earns even less than one who signals a non-copartisan identity (whether it is true or not). The prevalence of suspicion, combined with the additional punishment exacted for presumed dishonesty, means that unequal treatment by signaled identity is reduced when falsification is allowed. In this setting the gap is in fact eliminated.

Participants’ open-ended explanations of their DG decisions provide face-value evidence that these decisions were occasionally motivated by considerations of partners’ honesty or dishonesty. When falsification is an option, a signal of copartisanship is occasionally read as a sign of dishonesty. An example comes from a strong Democrat in the falsification condition whose partner signaled they were a Democrat: “I worry that they changed just to hope to appeal to me feeling they were ‘like me’ and wanting to share. I resent this possibility and decided not to share.” Or, similarly from a Republican: “I do not believe that Player B told the truth I think changed their answer to get closer to mine…. I do not want to give anything to people who are my enemy essentially.” Among participants in the falsification condition whose partner was a signaled copartisan, 13.33% referenced dishonesty in their open responses. This is greater than the share (0.00%) who used similar language when paired with a signaled non-copartisan in the baseline condition (*P* < 0.001, one-sided t-test, H_1_: *μ*_1_ > *μ*_2_) or the share (0.83%) who used similar language when paired with a signaled non-copartisan in the non-disclosure condition (*P* < 0.001, one-sided t-test, H_1_: *μ*_1_ > *μ*_2_).

Why do some participants in the falsification condition believe a signaled copartisan is a copartisan whereas others believe a signaled copartisan is a non-copartisan? Part of the answer lies in the incentives induced by a participant’s own identity. The stronger a participant’s partisan affiliation, the greater might be the reward for a partner who identifies as a copartisan, the greater might be the punishment for a partner who identifies as a non-copartisan, or both. For example, compared to a participant who identifies as a “Not very strong Democrat,” a participant who identifies as a “Strong Democrat” should think a Republican partner has a stronger incentive to falsify. To explore this, we model the belief that a partner is a non-copartisan as a function of a participant’s partisanship strength, among participants in the falsification condition whose partners signaled copartisanship (S22 Table in S1 File). Results suggest that a one-point increase in a participant’s partisanship strength corresponds to a 7.15% increase in the predicted probability of believing a signaled copartisan is a non-copartisan (*P* < 0.01). Note, however, that this association should be interpreted cautiously, because we did not manipulate participants’ partisanship strength. It is possible, for example, that strong partisans are more skeptical than other participants; this could partially account for the aggregate reduction in unequal treatment under the falsification condition.

By contrast, an exact match between a participant’s affiliation and their partner’s signaled affiliation does not predict suspicion (S23 Table in S1 File). Neither does the distance between a participant’s affiliation and their partner’s signaled affiliation (on the six-point scale) (S24 Table in S1 File).

### Non-disclosure

Why do participants exact a penalty for presumed dishonesty, above and beyond the one exacted for simply being a non-copartisan? One straightforward possibility is that people punish others when they lie for undeserved gain. However, they may punish others not only for presenting a “false” identity, but because they fail to present their “true” identity, i.e., for not reciprocating the disclosure of another person’s identity in an information exchange. To examine this possibility, we look to the non-disclosure condition, and specifically, the sub-condition in which partners withheld their party affiliation from participants.

Participants’ open-ended explanations do suggest some were piqued by a partner’s decision to withhold their affiliation after having learned the participant’s. As one not very strong Democrat put it, “I think that player B should have shared their information with me as they had information about me already. Since B did not want to share I felt slighted so did not share much money.” Occasionally, participants read the decision to withhold as a sign of mistrust in the participant and, by extension, the participant’s copartisans. One strong Democrat explained: “I would have sent them half if they had revealed their answer, no matter what it was. But since they hid it, I’m assuming they are a Republican who thinks I’ll punish them for not believing the same things as me. So, paradoxically and someone [sic] counterintuitively, I am instead punishing them for not trusting me to have good intentions.”

But do contributions themselves support the notion that participants punish partners for failing to reciprocate the disclosure of the participant’s affiliation? If so, participants in the non-disclosure condition whose partners withheld should behave less equitably than participants in the baseline condition whose partners signaled non-copartisanship. Among participants in the non-disclosure condition whose partner withheld their affiliation, 34.94% behaved equitably. This is slightly less than the 37.34% who behaved equitably toward a signaled non-copartisan in the baseline condition. However, the difference is not statistically significant (*P* = 0.306, one-sided t-test, H_1_: *μ*_1_ < *μ*_2_).

Moreover, participants were less generous to partners whom they suspected of lying about their affiliation than to partners who withheld their affiliation. Recall that in the falsification condition just 23.92% of participants behaved equitably toward a partner who signaled they were a copartisan but whom participants believed to be a non-copartisan. This is less than the 34.94% of participants who behaved equitably toward a partner who withheld their affiliation in the non-disclosure condition (*P* < 0.05, one-sided t-test, H_1_: *μ*_1_ < *μ*_2_).

Together, the findings suggest participants punish suspected liars not for failing to disclose their true identity, but for (presumably) disclosing a false identity for undeserved gain.

## Discussion

When others can neither withhold nor falsify their partisanship, people are more generous to those who signal copartisanship than to those who signal non-copartisanship. When others can falsify their partisanship, the gap in generosity to those who signal copartisanship versus non-copartisanship is reduced. In the setting of this experiment, it is eliminated. In sum, the availability of falsification reduces unequal treatment by signaled identity, and this reduction does not come at the expense of overall contributions. Unequal treatment is reduced in part because participants—especially strong partisans—believe some partners who signal a copartisan identity are in fact non-copartisans. Nevertheless, as an individual strategy, falsification is risky: some of those who signal copartisanship are suspected of dishonesty and punished severely.

The punishment exacted on those suspected of dishonesty exceeds the punishment exacted on those who signal an outgroup identity. Why? We examined whether and how much participants punished partners who withheld their identity, thereby failing to reciprocate the disclosure of the participant’s own identity. Partners who withheld their identity were not punished above and beyond those who signaled that they were non-copartisans. In addition, they were punished less than partners who signaled that they were copartisans but were suspected of being non-copartisans. In sum, people punish specifically for presenting false information, not for failing to present true information.

Even in the wake of growing fluidity around gender and, by some accounts, race [51, 52], signals of these and other identities are neither easy to conceal nor modify. Identities that can be more readily misrepresented can also serve as potent bases of discrimination when they are reliably signaled [33–35, 53]. This is true not only for partisanship, but also for other divisive, concealable identities like religion, immigration or citizenship status, sexual orientation, and social class. For these identities, falsification and non-disclosure may promote the “self-fulfilling illusion” that one’s social ties are homogeneous, politically or otherwise [20, 21, 54]; but see [55]. The possibility for misrepresentation might therefore seem to undermine a healthy culture of debate by contributing to echo chambers (see [28]). More optimistically, though, the uncertainty that stems from misrepresentation, and falsification specifically, could reduce unequal treatment across group lines.

Our findings mirror the real-world deployment of large-scale misrepresentation to protect those who hold stigmatized but concealable identities. Following the proposed addition of a citizenship question to the 2020 US Census, for example, some activists called on both citizens and non-citizens to boycott the question (e.g., [56]). Our findings are also consistent with work in which inequality is reduced when a concealable trait, like wealth, is made invisible (in this case, by the researchers) [57].

Several points, however, warn against a hasty endorsement of practices that enable misrepresentation or, like the US military’s former “don’t ask, don’t tell” policy, mandate it. First, we need to know whether some groups are bearing an undue burden by assessing whether the availability of misrepresentation also reduces unequal treatment in terms of real, as opposed to signaled, identities. This question, which is beyond the scope of this study, is ripe for further research. Second, the loftier goal would be to reduce unequal treatment even when concealable identities are known with certainty.

## Materials and methods

The study was approved by New York University Abu Dhabi’s Institutional Review Board, and the design and analysis were registered through Experiments in Governance and Politics (egap.org, 20190722AB) prior to the analysis of outcome data. The experiment did not involve deception, and participants’ responses are anonymous.

The data and code necessary to reproduce the analyses reported in this paper are available on Datavarse (thedata.org). This section provides additional information regarding (1) the inclusion criteria for the experiment; (2) the inclusion criteria for the analytic sample; (3) participant compensation; (4) the weights used.

### Inclusion criteria for the experiment

We took measures to ensure participants (1) entered the study just once, (2) were adults living in the United States, (3) identified as Democrats or Republicans, (4) were likely to provide high-quality responses, (5) formed roughly equal-sized groups across conditions, and (6) understood the incentive structure of the experimental game. Some of these requirements were communicated in the consent form. Additional details on all of the measures we took are outlined in the S1 File. Here, we focus on how we solicited party identification, because assignment to treatment was related to that.

Party identification was first solicited using the item: “Generally speaking, do you consider yourself a Republican, an Independent, a Democrat or something else?” Answer choices included “Republican,” “Independent,” “Democrat,” and “Something else.” People who identified as “Independent” or “Something else” were directed to an end-of-survey message and prevented from completing the study. Those who identified as “Republican” or “Democrat” were directed to a follow-up item about their party identification strength, solicited using the item: “Where would you put yourself on this scale?” Answer choices include “Strong Republican,” “Republican,” “Not very strong Republican,” “Not very strong Democrat,” “Democrat,” and “Strong Democrat.” Participants were assigned to a condition that had not yet met the quota for participants of that party-strength. Once we had enough participants of that party-strength across all conditions, a prospective participant was not allowed to complete the study.

### Inclusion criteria for the analytic sample

We excluded additional participants from our analytic sample to ensure data quality. First, we excluded 11 participants who gave conflicting responses to the first and second party identification items (for example, a “Democrat” who later identified as a “Strong Republican”). In addition, we excluded 302 participants who incorrectly answered an attention check item that appeared after the DG. Specifically, the item assessed what party-strength participants learned for their partner (a “None of the above” option was intended for participants whose partners withheld their partisanship).

We also asked participants what they thought their partners’ party-strength really was, anticipating that some participants would not believe the party-strength partners reported about themselves. We excluded 130 participants from the baseline and non-disclosure conditions whose responses were theoretically unanticipated, indicating they did not take up treatment. This includes, for example, participants in the baseline condition who thought that a partner who signaled a copartisan identity was, in fact, a non-copartisan. We evaluate the robustness of our results against this decision in the S1 File, and find no substantive differences. Participants excluded from the analytic sample met one or more of the exclusion criteria described. In sum, we follow suggestions to analyze the subsample of compliant and attentive participants [58].

### Compensation

Participants who failed the demographic screening questions received $0.05 for their time. Additionally, participants who twice failed one or more comprehension check questions were directed to an end-of-survey message and they received $0.10 for their time. These amounts were distributed via compensation HITs targeted to the relevant MTurk workers. Participants who completed the survey received a $0.50 show-up fee, regardless of their DG decision. In addition, they received the portion of the $2.00 endowment they retained, as a bonus. This was, on average, $1.47. Finally, all participants were awarded a $0.20 bonus, the amount they were told they would receive if they correctly guessed their partner’s basic demographic characteristics. In sum, on average, participants who completed the study earned $2.17. On average, participants in the analytic sample completed the survey in 7.85 minutes, for an average hourly rate of $16.59.

### Weighting

Analyses reported in the main text give equal weight to each party-strength category, ensuring that the results do not hinge on the relative numbers of participants in each sub-condition. Specifically, we assigned the same weight to each participant in the same party-strength category, such that the sum of the weights for each category equals one-sixth of the analytic sample. These weights range from 0.79 to 1.54. The analyses reported in “Patterns of beliefs,” are an exception: these analyses are not weighted, as they also concern participants who did not take up treatment, i.e., whose beliefs were theoretically unanticipated.

In the S1 File, we also report analyses based on an alternative weighing strategy, one in which we weight each observation so the sample resembles (in terms of educational attainment, income, race/ethnicity, age, and gender) a representative sample of US Americans who identify as Democrats or Republicans. The representative sample comes from the 2018 Cooperative Congressional Election Survey (CCES). These weights were created using the anesrake(.) function in the in R package anesrake. We capped weights at 5. Weighting proceeded in two steps: we created weights separately for Republicans and Democrats to match the characteristics of their corresponding representative samples. As a result of rebalancing, no characteristic of the weighted sample differs from the CCES sample by more than 0.020 in terms of standardized mean difference. In the second step, we adjusted the weights so that Republicans and Democrats each accounted for half of the sample. Finally, we also report the results from Table 2, unweighted, in S25 Table in S1 File; results are substantively similar.

## Supporting information

S1 File (PDF)
