# Correction: Unequal treatment toward copartisans versus non-copartisans is reduced when partisanship can be falsified

**DOI:** 10.1371/journal.pone.0264248

**Published:** 2022-02-15

**Authors:** Maria Abascal, Kinga Makovi, Anahit Sargsyan

The following information is missing from the Funding statement: This study was supported by NYUAD Center for Interacting Urban Networks (CITIES), funded by Tamkeen and by the Swiss Re Institute under the Quantum Cities™ initiative (Award #: CG001).
