# Supplementary material for: Unequal treatment toward copartisans versus non-copartisans is reduced when partisanship can be falsified
Source: PLoS One. 2021 Jan 27;16(1):e0244651. doi: 10.1371/journal.pone.0244651 (PMC7840019; doi:10.1371/journal.pone.0244651)
Supplement: S1 File — (PDF) [file pone.0244651.s001.pdf]

S1 File: Unequal treatment is reduced when group identity can be falsified

Maria Abascal, Kinga Makovi, Anahit Sargsyan

1 Analyzing the impact of falsification on unequal treatment

In this section we derive the possible effects of falsification on unequal treatment across signaled identities. We depart from the assumption that signaled partisanship is uniformly believed. In the real world, people may not believe the information they receive about others' political views. Their actions may be determined by both the signal people receive and whether they believe it.

The parameters that determine unequal treatment when information about one's interaction partner is truthful, complete, and believed are:

$$F_{\text{co}} = \text{the share of fair contributions to copartisans} \tag{1}$$

$$F_{\text{non-co}} = \text{the share of fair contributions to non-copartisans} \tag{2}$$

Unequal treatment, as defined in the paper, represents the difference in contributions: $\Delta = F_{\text{co}} - F_{\text{non-co}}$, which tells us how much more likely it is that a signaled co-partisan receives a fair share than a signaled non-copartisan.

With the availability of falsification, signaled copartisans and signaled non-copartisans may or may not be believed. The share of fair contributions may then be expressed as a function of

the share of fair contributions under truthful, complete, and believed information.

$$F_{\text{co}}^{\text{believed}} = F_{\text{co}} + \alpha \tag{3}$$

$$F_{\text{co}}^{\text{disbelieved}} = F_{\text{co}} + \beta \tag{4}$$

$$F_{\text{non-co}}^{\text{believed}} = F_{\text{non-co}} + \gamma \tag{5}$$

$$F_{\text{non-co}}^{\text{disbelieved}} = F_{\text{non-co}} + \delta \tag{6}$$

α is the deviation from the probability of a fair contribution when one meets a signaled copartisan whom one believes. If α is 0, the signaled and believed copartisan is neither rewarded nor punished compared to baseline. Theoretically, it makes sense to assume that α is non-negative. Negative values would be counter-intuitive, as they would mean a signaled copartisan who is believed to be a copartisan is punished.

β is the deviation from the probability of a fair contribution when one meets a signaled copartisan whom one does not believe. Theoretically, it makes sense to assume that β is not positive, i.e., that a signaled copartisan who is believed to be a non-copartiasn is not rewarded.

γ is the deviation from the probability of a fair contribution when one meets a signaled non-copartisan whom one believes. Theoretically, γ may be either positive and negative. A positive γ could be read as a reward for honesty; a negative γ could be read as a punishment for insolence.

Finally, δ is the deviation from the probability of a fair contribution when one meets a signaled non-copartisan whom one does not believe. Theoretically, δ is likely to be non-positive,i.e., a signaled non-copartisan who is not believed is not likely to be rewarded.

In sum, we have theoretical reasons to restrict the ranges of α (non-negative), β (non-positive) and δ (non-positive), but not γ.

Two final parameters are:

$$P_{\text{co-bel}} = \text{the probability that a signaled copartisan is believed} \tag{7}$$

$$P_{\text{non-co-bel}} = \text{the probability that a signaled non-copartisan is believed.} \tag{8}$$

It is plausible to think that $P_{\text{co-bel}}$ is smaller than 1, that is, that not all copartisans will be believed, and that $P_{\text{non-co-bel}}$ is 1. Note that our empirical data confirms this assumption, and while it is theoretically possible for non-copartisans not to be believed, they are, in fact, overwhelmingly believed.

From here, we may compute unequal treatment under falsification, and compare its value to the baseline.

Fair contributions to signaled copartisans in falsification:

$$F_{\text{co}}^{\text{fals}} = P_{\text{co-bel}} \cdot (F_{\text{co}} + \alpha) + (1 - P_{\text{co-bel}}) \cdot (F_{\text{co}} + \beta)$$

Fair contributions to signaled non-copartisans in falsification:

$$F_{\text{non-co}}^{\text{fals}} = P_{\text{non-co-bel}} \cdot (F_{\text{non-co}} + \gamma) + (1 - P_{\text{non-co-bel}}) \cdot (F_{\text{non-co}} + \delta)$$

The quantity of interest is:

$$\begin{aligned}
&\Delta^{\text{fals}} = \\
&\quad P_{\text{co-bel}} \cdot (F_{\text{co}} + \alpha) + (1 - P_{\text{co-bel}}) \cdot (F_{\text{co}} + \beta) - \\
&\quad [P_{\text{non-co-bel}} \cdot (F_{\text{non-co}} + \gamma) + (1 - P_{\text{non-co-bel}}) \cdot (F_{\text{non-co}} + \delta)],
\end{aligned}$$

which is

$$\begin{aligned}
&\Delta^{\text{fals}} = \\
&\quad P_{\text{co-bel}} \cdot (F_{\text{co}} + \alpha) + (1 - P_{\text{co-bel}}) \cdot (F_{\text{co}} + \beta) - \\
&\quad P_{\text{non-co-bel}} \cdot (F_{\text{non-co}} + \gamma) - (1 - P_{\text{non-co-bel}}) \cdot (F_{\text{non-co}} + \delta).
\end{aligned}$$

With further simplification:

$$\Delta^{\text{fals}} =$$

$$P_{\text{co-bel}} \cdot F_{\text{co}} + P_{\text{co-bel}} \cdot \alpha +$$

$$F_{\text{co}} + \beta - P_{\text{co-bel}} \cdot F_{\text{co}} - P_{\text{co-bel}} \cdot \beta -$$

$$P_{\text{non-co-bel}} \cdot F_{\text{non-co}} - P_{\text{non-co-bel}} \cdot \gamma -$$

$$F_{\text{non-co}} - \delta + P_{\text{non-co-bel}} \cdot F_{\text{non-co}} + P_{\text{non-co-bel}} \cdot \delta.$$

Collecting the parts that are a function of quantities previously known:

$$\Delta^{\text{fals}} =$$

$$F_{\text{co}} \cdot (1 + P_{\text{co-bel}} - P_{\text{co-bel}}) +$$

$$F_{\text{non-co}} \cdot (P_{\text{non-co-bel}} - P_{\text{non-co-bel}} - 1) +$$

$$P_{\text{co-bel}} \cdot \alpha + \beta - P_{\text{co-bel}} \cdot \beta - P_{\text{non-co-bel}} \cdot \gamma - \delta + P_{\text{non-co-bel}} \cdot \delta.$$

Finally, continuing simplification:

$$\Delta^{\text{fals}} =$$

$$\Delta +$$

$$P_{\text{co-bel}} \cdot (\alpha - \beta) +$$

$$P_{\text{non-co-bel}} \cdot (\delta - \gamma) +$$

$$\beta - \delta.$$

From this derivation, we express the relationship between unequal treatment in baseline (when signals are truthful and believed with no possibility of falsification), and unequal treatment in falsification (when signals are possibly false, and therefore may not be believed). Whether unequal treatment increases, decreases, or stays the same depends on the sign of $P_{\text{co-bel}} \cdot (\alpha - \beta) + P_{\text{non-co-bel}} \cdot (\delta - \gamma) + P_{\text{co-bel}}$.

Because there are six parameters in this expression, we start by analyzing it with an additional and plausible simplifying assumption: all non-copartisans are believed, $P_{\text{non-co-bel}} = 1$. In this case, we end up with $P_{\text{co-bel}} \cdot (\alpha - \beta) + \beta - \gamma$, i.e., only four parameters.

Unequal treatment increases when:

$$P_{\text{co-bel}} \cdot (\alpha - \beta) + \beta - \gamma > 0$$

The first term in this expression, $P_{\text{co-bel}} \cdot (\alpha - \beta)$, will be non-negative, because we assumed that α is non-negative, β is non-positive, and $P_{\text{co-bel}}$ is positive. However, the second term, $\beta - \gamma$ has an unclear sign. We assumed that β is non-positive, but γ is not restricted. The relative magnitudes of the first and second expressions are also unknown. For instance, the following plausible parameters would produce increased unequal treatment: $P_{\text{co-bel}} = 0.8$, i.e., when 80% of signaled copartisans are believed, when α is 0 (no reward for being honest for a copartisan), we arrive at the following expression $0.2 \cdot \beta > \gamma$. **When non-copartisans are rewarded for honesty, i.e., γ is positive, unequal treatment cannot increase**. However, when non-copartisans are punished for insolence, e.g., when $\gamma = -0.05$, then, for instance with $\beta = -0.1$, i.e., with a larger punishment for lying than that for insolence, the expression of $-0.02 = 0.2 \cdot -0.1 > -0.05$ holds.

Unequal treatment decreases when:

$$P_{\text{co-bel}} \cdot (\alpha - \beta) + \beta - \gamma < 0$$

The following plausible parameters would yield increased unequal treatment: $P_{\text{co-bel}} = 0.8$, i.e., when 80% of signaled copartisans are believed, when α is 0 (no reward for being honest for a copartisan), we arrive at the following expression $0.2 \cdot \beta < \gamma$. In this case, **when non-copartisans are rewarded for honesty, i.e., γ is positive, unequal treatment decreases** with all plausible values of β. When non-copartisans are punished for insolence, for instance when $\gamma = -0.05$, and $\beta = -0.3$ (a plausibly larger punishment for lying than for insolence), then the

condition is still met $-0.06 = 0.2 \cdot -0.3 < -0.05$. I.e., unequal treatment may decrease with both a reward for honesty, as well as a punishment for insolence, depending on the values of the other parameters.

In sum, with plausible assumptions, the direction in which unequal treatment changes over baseline hinges on the values of the following parameters: (1) the propensity to believe an alter who signals an ingroup affiliation, (2) the reward for signaling an ingroup affiliation when that affiliation is believed, (3) the punishment for signaling an ingroup affiliation when that affiliation is not believed, and (4) the reward for signaling an outgroup affiliation.

2 Extended description of data collection

Data collection took place between July 10 and 20, 2019. Participants were recruited through Amazon Mechanical Turk (MTurk) via the TurkPrime platform. The study was approved by New York University Abu Dhabi's Institutional Review Board, and the design and analysis were registered through Experiments in Governance and Politics (egap.org, 20190722AB) prior to the analysis of outcome data. The experimental procedure is presented in Fig. S1, and screening points are discussed in detail below.

Inclusion criteria in the survey experiment

Screens for duplicate responses

We used multiple screens to avoid duplicate responses. First, we created a survey group within TurkPrime for all HITs associated with this study. The same MTurk worker cannot take multiple surveys in the same TurkPrime survey group. Second, we activated the "ballot box stuffing" option within Qualtrics (where we programmed the survey) to prevent multiple entries from the same IP address. Regardless, on the first day of fielding, we encountered multiple workers who started the survey multiple times, including workers who had been screened out based on their initial responses to the demographic items or the comprehension check items. To deal with this, we sequentially compiled a list of the MTurk IDs of workers who entered the survey using an external SQL database. And, we automatically verified that each new worker was not already on this list. Our sample is limited to unique participants who took the study once and who saw instructions for just one experimental condition. This database was responsible for the first screening point in the flowchart.

Data quality

We took several measures to ensure data quality. Only MTurk workers who were 18 years or older and who were located in the United States—as specified on their MTurk account and by their IP address—could see the "Human Intelligence Task" (HIT). To be eligible, workers also needed to have at least 100 HITs approved and a 95% approval rate. We also excluded workers from suspicious geolocations and those on the "universal exclude list," both managed by TurkPrime.

Screens for demographic characteristics and party affiliation

Participants first answered questions about their basic demographics, including race/ethnicity, age, sex, state-of-residence, and party identification. Participants who reported that they were under 18, those who reported that they lived in "Some other country," and those who did not identify as "Democrat" or "Republican" were not allowed to complete the study. They received $0.05 for their time via a compensation HIT. These screens are represented by the second screening point in the flowchart.

We aimed to recruit roughly equal numbers of participants within each of the six points on the party identification scale. We also aimed to assign roughly equal numbers of participants to each experimental condition. To this end, we screened out MTurk workers who exceeded programmed quotas within their party-strength–condition combination. These workers were not allowed to complete the study; they received $0.05 for their time via a compensation HIT. We initially set quotas to 30, then reduced them to 25 over the last three days of data collection to deal with the under-representation of certain party-strengths among MTurk workers. This step is represented by the third screening point in the flowchart.

To ensure our survey was effectively reaching MTurk workers who were still eligible to take it, on the second-to-last day of data collection, we restricted the HIT to workers who did

not identify as “Very liberal,” and on the last day, we restricted the HIT to workers who do not identify as “Very liberal” or “Liberal.” These screening criteria were available through TurkPrime. We implemented these restrictions to target self-identified Republicans, very few of whom identify as “Very liberal” or “Liberal” in the 2018 Cooperative Congressional Election Survey (CCES) (*1*).

Screens for comprehension of the experimental game

Participants who were not screened out then reviewed instructions for a dictator game (DG), which they played with another MTurk worker. Interaction partners were recruited in a separate data collection, as part of the same experiment. The results of the other data collection are discussed in other work. Our DG was a simple, one-shot game in which Player A received $2.00 and could share any portion of that endowment with Player B. In the first data collection, which is the subject of this paper, all participants were assigned to the role of Player A. Before making their decision, participants answered three comprehension check questions about the game. They were allowed to reread the instructions before answering these questions. Those who failed twice to answer all three questions correctly were directed to an end-of-survey message and were prevented from completing the study or reentering the study, as detailed above. They received $0.10 for their time via a compensation HIT. This is the last screening point represented in the flowchart.

5,825 unique MTurk workers opened the link to the survey. Of these, 243 left the survey due to connectivity issues or because they decided to withdraw from participation. An additional 1,819 were screened out based on their responses to the initial demographic questionnaire, 568 were screened out because they exceeded programmed quotas for their party-strength–condition combination, and 264 were screened out because they failed twice to answer all comprehension check questions correctly. Our base sample includes 2,931 unique MTurk workers who com-

pleted the experiment.

Our analytic sample is defined by additional restrictions meant to enhance data quality. These are detailed in "Materials and Methods." In review, our analytic sample is limited to those 2,538 participants who correctly answered an attention-check question and who took up treatment. Tab S1 breaks down our analytic sample by experimental arms and participants' party-strength. Tab S2 breaks down the analytic sample by participants' party-strength and the information they received about their interaction partners. Note that Democrats are over-represented in our sample, as they are on MTurk (*2*).

3 Justifications for allocation decisions in the DG

After making their DG decision, participants were asked to describe how they made their decision. Participants were not allowed to proceed to the next screen until they typed some text in the response box. Responses ranged from one word to detailed, multi-sentence entries. The average response contained 25.78 words (SD = 16.73). We read all of the responses to this question, and we developed the response codes described below. After we developed these categories, we asked two other researchers to code participants' qualitative responses, i.e., assign them to these categories. These researchers coded the qualitative responses separately then met to resolve inconsistencies. They were not familiar with the qualitative hypotheses registered and tested by the authors.

1. **Partisanship**: The participant gave this amount because their interaction partner signaled a certain political affiliation;

2. **My partisanship**: The participant gave this amount because of their own political affiliation;

3. **Extreme**: The participant gave this amount, or did not give a different amount, because their interaction partner assumed/did not assume a strong party-identification. Responses in this category are a subset of **Partisanship** responses;

4. **Fair**: The participant gave this amount because they wanted to act fairly;

5. **Selfish**: The participant gave this amount to maximize their payoff;

6. **Reciprocity**: The participant gave this amount because this is what others did/not give them in a previous experiment, or because this is how they expect to be treated if roles were reversed;

7. **Merit**: The participant gave this amount because Player B worked on the same task, and should be compensated for their time/participation;

8. **Ethics**: The participant gave this amount because "it was the right thing to do." Answers referencing morality or kindness, but which did not explicitly reference fairness or reciprocity, fall in this category;

9. **No-matter-what**: The participant gave this amount despite/regardless of the political affiliation of their interaction partner. Some stated that they made their contribution decision before they learned their interaction partner's party-strength;

10. **Don't know B**: The participant gave this amount because they do not know who their interaction partner personally;

11. **Dishonesty**: The participant gave this amount because the other person may have changed/compromised their answer;

12. **Honesty**: The participant gave this amount because the other person likely did not change/compromise their answer;

13. **Hardship**: The participant gave this amount because they are facing (financial) hardship;

14. **Self-presentation**: The participant gave this amount in order to present themselves in a certain way, sometimes with the intent of not alienating their partner.

These categories are non-exclusive. The average participant gave 1.55 reasons (SD = 0.83) for their allocation decision. The breakdown of reasons by experimental condition is presented in Tab S3.

4 Main analyses disaggregated by participant partisanship

Tabs. S7 and S8 report the same information as Tab. 2, disaggregated by participant's partisanship. Similarly, Figs. S2 and S3 report the same outcomes as Fig. 1, separately for Democrats and Republicans. The qualitative patterns in these data are similar across parties. Note, however, that the weights used in these analyses have been recalculated and reflect the true sample size of Republicans and Democrats, while all three party-strength categories are given equal weight. Consistent with the over-representation of Democrats on MTurk, comparisons are better powered for this group.

5 Main analyses replicated with CCES weighting

Fig. S4 reports the same analyses as Fig. 1, but assigning weights to participants such that Democrats and Republicans, each, make up half of the sample, and they resemble, in terms of basic demographics, nationally representative samples of Democrats and Republicans, respectively. The results do not reveal substantive differences. In the baseline condition, however, the difference between contributions to copartisans and non-copartisans is not statistically significant at a conventional level. However, the decisions of a minority of our sample are weighted heavily in these analyses. For example, among Democrats, just 123 individuals make up 47.24% of the weighted sample, whereas among Republicans, just 195 individuals make up 76.39% of the weighted sample (Tab. S11).

6 Main analyses replicated with contributions

Based on the distribution of contributions, we chose to analyze the share of equitable contributions. Fig. S5 instead reports contributions in US dollar amounts. The substantive results from this analysis resemble those presented in Fig. 1.

7 Main analyses replicated by dropping participants who were skeptical about the existence of their partners

Here, we drop participants who expressed doubt about the existence of their partner. Participants were able to express doubt in two, open-ended questions. In the first, we asked them to explain their DG decision. In response to this question, for example, one participant wrote, “The other person is not real and I want to maximize my bonus.” The second question asked participants if they found anything strange or surprising about the study. Some participants reported that they did not believe their partner was a real person. For example, one participant wrote, “I have doubts that the other participant is a real person.” In total, 213 participants expressed doubt in response to one or both of these questions. After excluding these participants, we recalculated the weights, because these skeptics were not equally distributed across the six party-strength categories, though the shares across party-strength were generally similar, ranging from 7.8% to 9.8%.

8 Beliefs about partners' demographic characteristics by partisanship, across experimental conditions

After participants made their DG decisions, they were asked a series of follow-up questions about their partner. This task was introduced using the following prompt: "*In this part of the study, we will ask questions about the other person in the activity you just did. It might be difficult to evaluate this person based on the limited information we gave you. Please do your best to answer each question, even if you do not feel like you have all of the information you need. If you answer all three questions correctly, you will earn an additional bonus of $0.20.*" Participants were thereby incentivized to report their best guesses about their partners. Specifically, we asked about their partner's gender, their educational attainment, and their race/ethnicity. The answer categories were identical to those participants used to report their own demographics. Tabs. S16–S21 report participants' beliefs about partners' whom they believed to be Democrats or Republicans, respectively. These results reveal that experimental treatment was not associated with participants' perceptions of their partners' demographics, conditional on their partners' believed partisanship. The one exception is that participants who believed their partners were Republicans were 4 percentage points more likely to believe these partners were White in the falsification condition than in either the baseline or non-disclosure conditions. It is unclear, however, whether or how these beliefs would drive the main results.

9 Robustness to selection into the analytic sample, including those who did not take up the treatment in baseline and non-disclosure

In the baseline and non-disclosure conditions, we are able to identify participants who did not take up treatment. These are participants who correctly recalled the reported identity of their partner but who believed their partner identified with the other party. In the falsification condition we cannot separate these individuals from those who believed that their interaction partners were lying. Note that including these participants would not alter the results reported in Tab. 1 or Fig. 1, as these results are conditioned on beliefs. Only Tab. 2 changes when including participants who did not take up treatment. All statistical tests based on this table yield the same conclusions reported in the main text, see Tab. S26.

10 Predicting disbelief in a signaled copartisan in the falsification condition

What predicts whether a participant in the falsification condition thinks a partner who signaled a copartisan affiliation is in fact a non-copartisan? We explore three hypotheses:

Hypothesis 1. Suspicion is positively predicted by the strength of a participant's partisan affiliation (*strength*).

Hypothesis 2. Suspicion is positively predicted by a match between a participant's affiliation and a partner's affiliation (*match*).

Hypothesis 3. Suspicion is negatively predicted by the distance between a participant's affiliation and a partner's affiliation (*distance*).

Strength corresponds to the strength of a participant's partisan affiliation, where "Strong Democrat" and "Strong Republican" $= 2$, "Democrat" and "Republican" $= 1$, and "Not very strong Democrat" and "Not very strong Republican" $= 0$. *Match* corresponds to a match between a participant's identity and a partner's signaled identity on the six-point scale. For example, for a participant who identities as a "Strong Democrat," this variable takes a value of 1 for a partner who signals "Strong Democrat," and 0 otherwise. *Distance* represents the absolute difference between a participant's identity and a partner's signaled identity on the six-point scale. For example, for a participant who identifies as a "Strong Democrat," this variable takes a value of 0 for a partner who signals "Strong Democrat," 1 for a partner who signals "Democrat," and so on.

The regressions presented in Tabs. S22–S24 predict disbelief in a partner's signaled identity among those participants in the falsification condition whose partner signaled that they were a copartisan.

References

1. S. Kuriwaki, Cumulative CCES Common Content (2006-2018) (2018).

2. C. Huff, D. Tingley, *Research & Politics* **2** (2015).

Figures

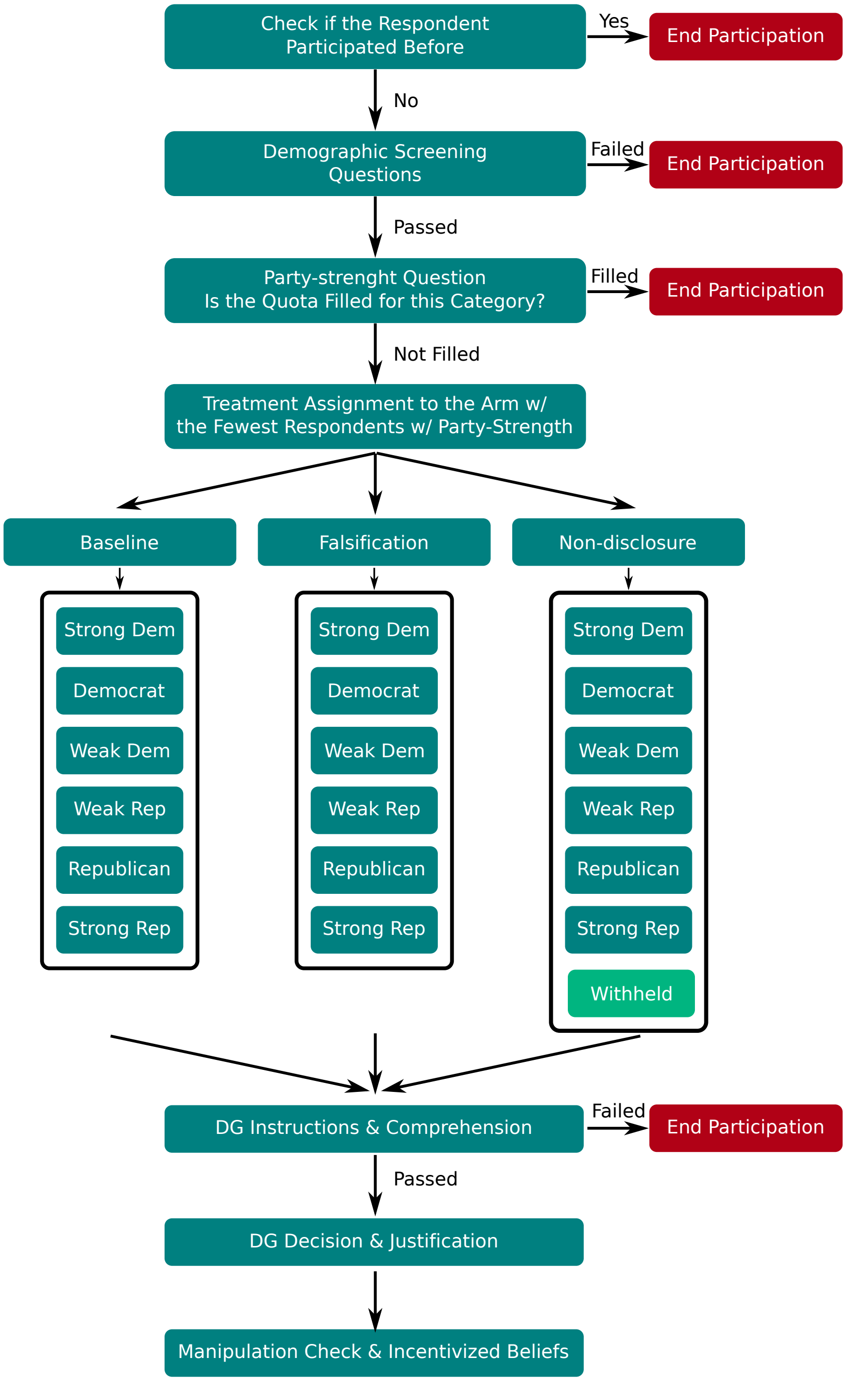

Figure S1: Flowchart of experimental procedures.

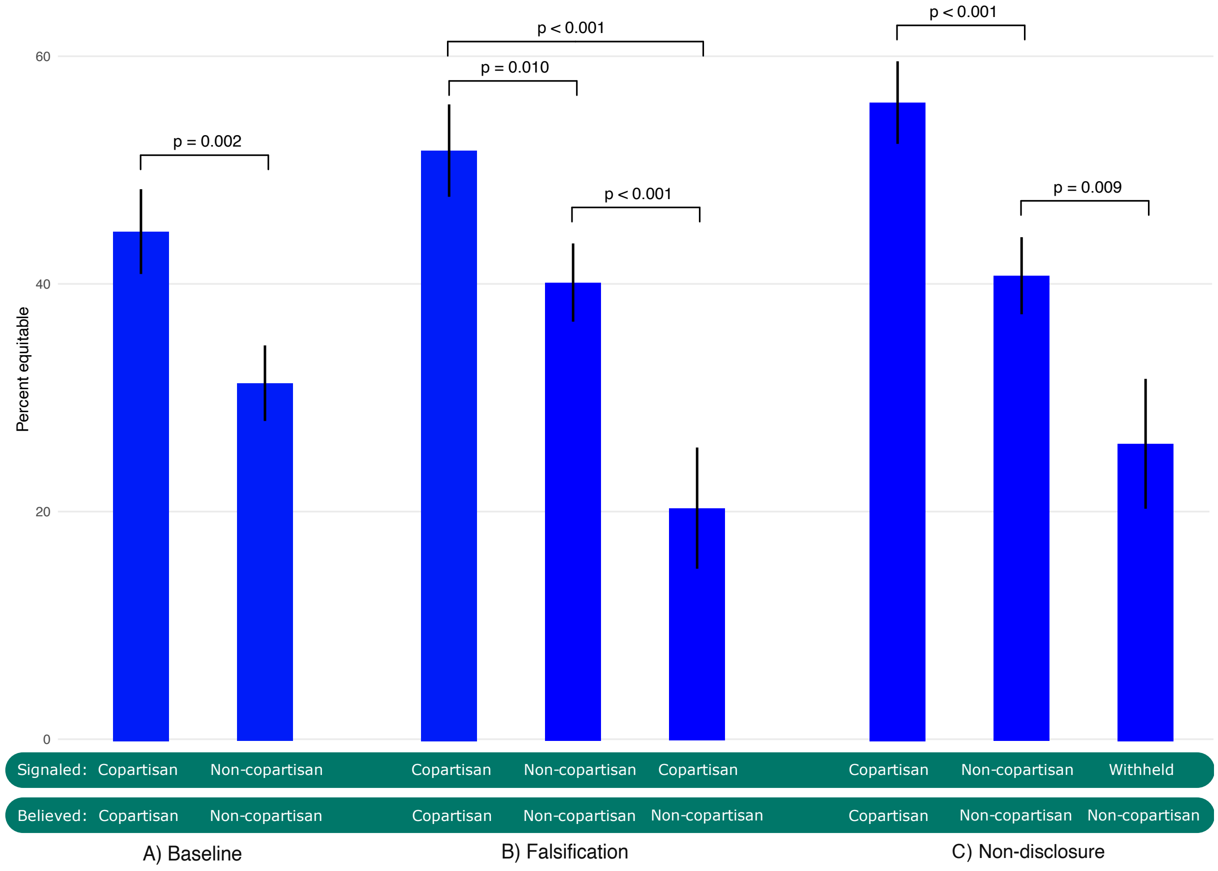

Figure S2: Share of equitable contributions from Democrat participants, by the identity signaled by partners and the identity believed by participants, across experimental conditions.

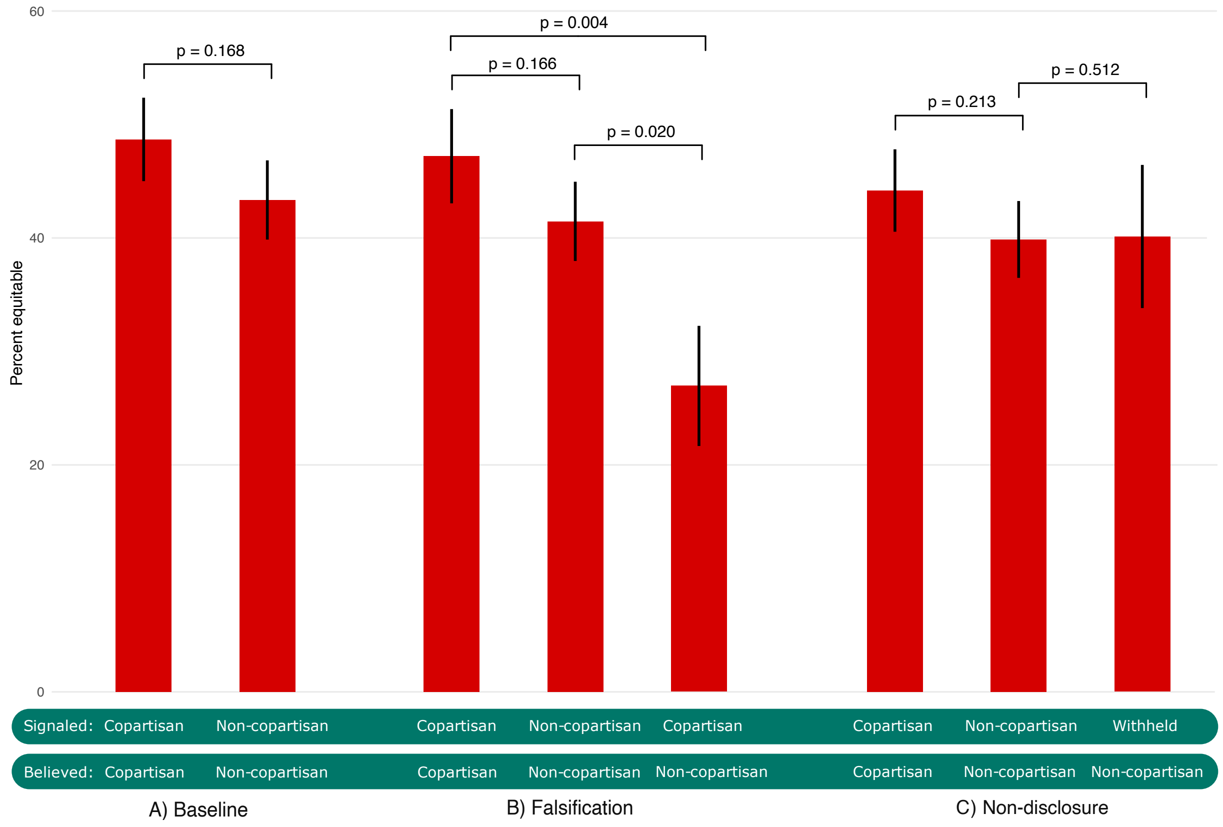

Figure S3: Share of equitable contributions from Republican participants, by the identity signaled by partners and the identity believed by participants, across experimental conditions.

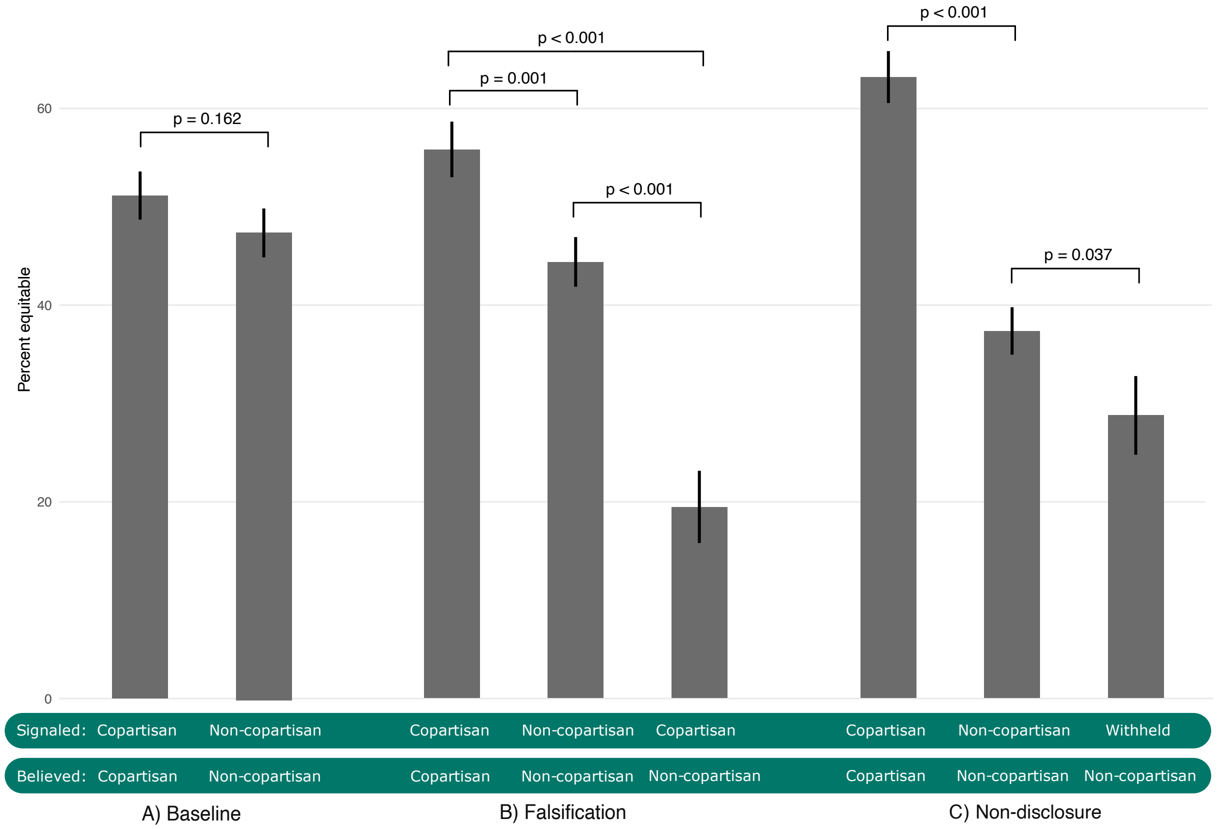

Figure S4: Share of equitable contributions by the identity signaled by partners and the identity believed by participants, across experimental conditions; CCES weighting.

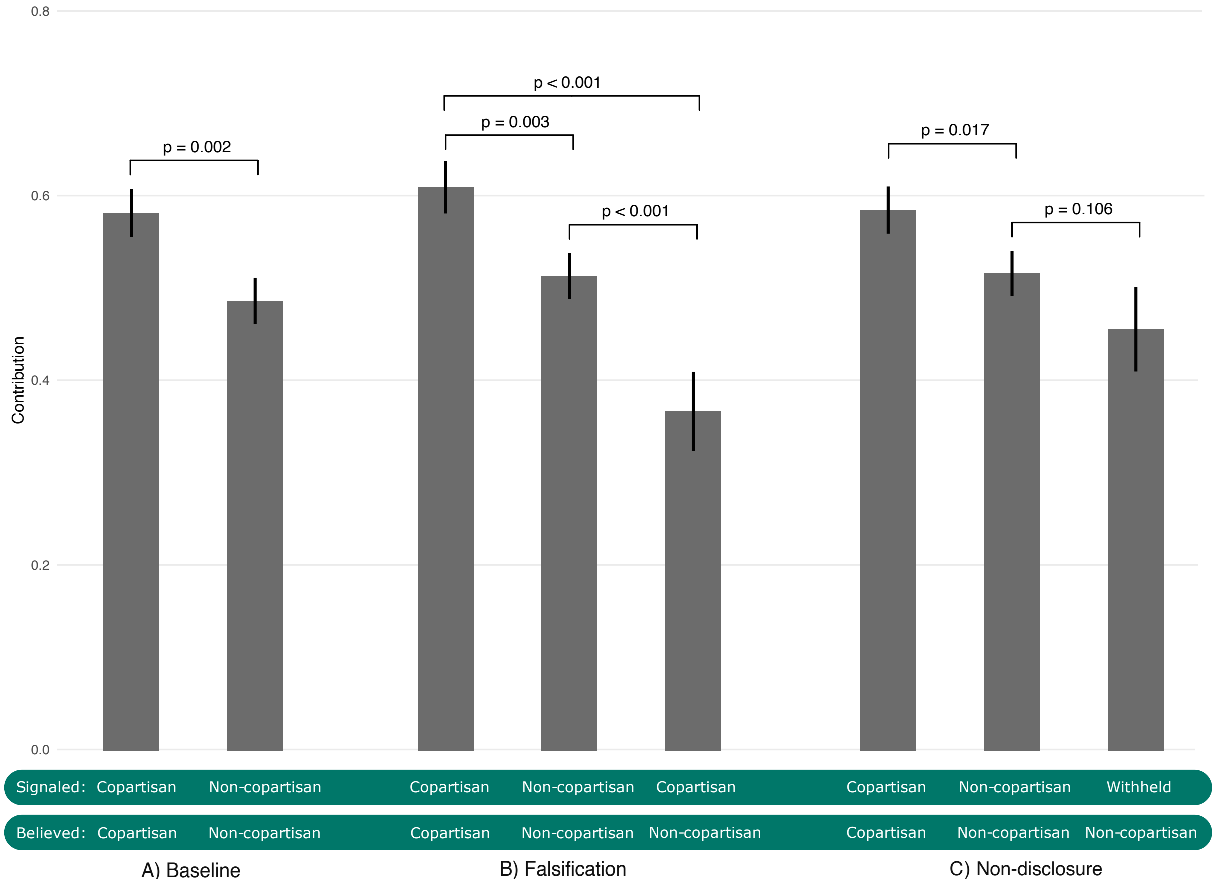

Figure S5: Contributions ($) by the identity signaled by partners and the identity believed by participants, across experimental conditions.

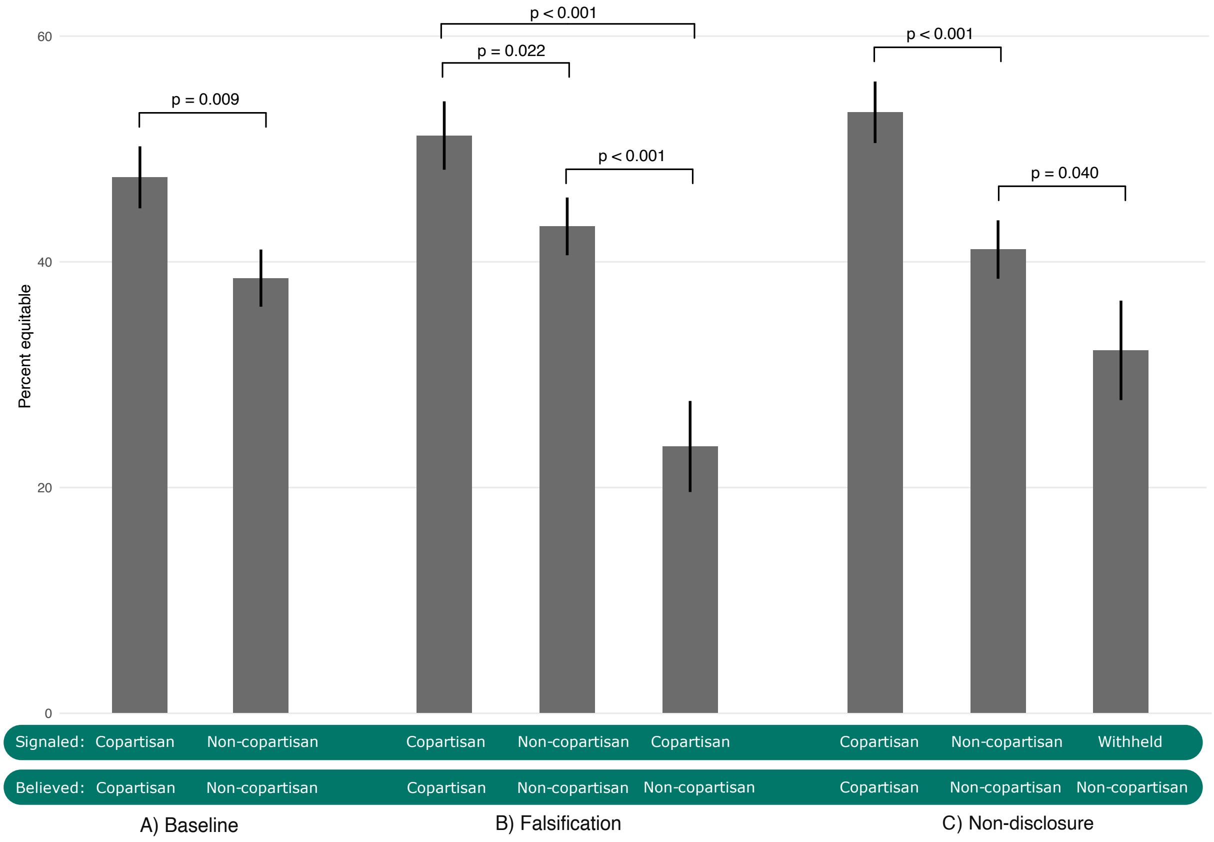

Figure S6: Share of equitable contributions from non-skeptical participants, by the identity signaled by partners and the identity believed by participants, across experimental conditions.

Tables

Dictators' party ID	Baseline	Non-disclosure	Falsification
Strong Democrat	166	192	175
Democrat	150	194	164
Not strong Democrat	122	161	144
Not strong Republican	113	146	124
Republican	127	152	136
Strong Republican	85	96	91

Table S1: Participants' party-strength by experimental condition

	Recipient's party ID shown to Dictator						
	Strong Democrat	Democrat	Not strong Democrat	Not strong Republican	Republican	Strong Republican	Undisclosed
Strong Democrat	84	80	82	85	80	94	28
Democrat	81	87	72	81	79	81	27
Not strong Democrat	63	66	59	72	67	74	26
Not strong Republican	56	63	66	58	56	63	21
Republican	63	72	66	64	64	62	24
Strong Republican	45	39	47	44	39	44	14

Table S2: Participants' party-strength by partners' reported party-strength.

Justification	Baseline (%)	Non-disclosure (%)	Falsification (%)
**Partisanship**	22.54	16.05	18.35
**My partisanship**	0.66	1.38	0.60
**Extreme**	1.44	2.02	1.56
**Fair**	39.45	41.66	37.17
**Selfish**	23.46	21.68	21.22
**Reciprocity**	13.24	12.75	8.99
**Merit**	8.39	6.70	8.51
**Ethics**	5.77	6.70	6.12
**No-matter-what**	21.10	26.99	25.78
**Don't know B**	2.88	4.14	4.44
**Dishonesty**	0.00	5.31	7.43
**Honesty**	0.00	1.59	0.48
**Hardship**	11.01	11.26	11.15
**Self-presentation**	2.36	1.17	1.08

Table S3: Justifications given for DG contributions across experimental conditions.

N	**Baseline** 924	**Non-Disclosure** 1080	**Falsification** 916
Age (mean (SD))	38.52 (12.68)	38.34 (12.13)	38.21 (11.92)
Female (mean (SD))	0.56 (0.50)	0.55 (0.50)	0.57 (0.50)
Not female (mean (SD))	0.44 (0.50)	0.45 (0.50)	0.43 (0.50)
White (mean (SD))	0.76 (0.42)	0.77 (0.42)	0.75 (0.43)
Non-White (mean (SD))	0.24 (0.42)	0.23 (0.42)	0.25 (0.43)
HS or less (mean (SD))	0.11 (0.32)	0.09 (0.28)	0.09 (0.29)
Some college (mean (SD))	0.34 (0.47)	0.33 (0.47)	0.34 (0.47)
Bachelor's degree (mean (SD))	0.39 (0.49)	0.44 (0.50)	0.41 (0.49)
Graduate or professional degree (mean (SD))	0.16 (0.37)	0.15 (0.36)	0.16 (0.37)
Less than $10,000 (mean (SD))	0.04 (0.19)	0.03 (0.17)	0.03 (0.18)
$10,000–$19,999 (mean (SD))	0.07 (0.25)	0.06 (0.24)	0.05 (0.23)
$20,000–$29,999 (mean (SD))	0.11 (0.31)	0.11 (0.31)	0.09 (0.29)
$30,000–$39,999 (mean (SD))	0.11 (0.31)	0.11 (0.32)	0.11 (0.31)
$40,000–$49,999 (mean (SD))	0.09 (0.29)	0.12 (0.32)	0.11 (0.31)
$50,000–$59,999 (mean (SD))	0.11 (0.31)	0.10 (0.30)	0.11 (0.31)
$60,000–$69,999 (mean (SD))	0.09 (0.29)	0.09 (0.29)	0.09 (0.29)
$70,000–$79,999 (mean (SD))	0.09 (0.28)	0.08 (0.27)	0.07 (0.26)
$80,000–$89,999 (mean (SD))	0.06 (0.23)	0.06 (0.23)	0.06 (0.25)
$90,000–$99,999 (mean (SD))	0.06 (0.23)	0.05 (0.22)	0.05 (0.23)
$100,000–$149,999 (mean (SD))	0.11 (0.32)	0.12 (0.32)	0.13 (0.34)
$150,000 and above (mean (SD))	0.05 (0.22)	0.05 (0.22)	0.05 (0.23)
Prefer not to say (mean (SD))	0.02 (0.12)	0.02 (0.15)	0.02 (0.15)
Failed attention check (mean (SD))	0.14 (0.34)	0.09 (0.28)	0.09 (0.29)
Failed treatment takeup (mean (SD))	0.09 (0.29)	0.08 (0.27)	–

Table S4: Descriptive statistics of participant demographics across experimental conditions. Note that participants had the option to select "male," "female," or "something else" for gender. Here, we collapsed participants who identified as "male" or "something else" into one category for those who identified as "not female." Note also that, regarding race/ethnicity, we categorized as Non-White all participants who selected a category other than White. In sum, Non-Whites include participants who identified as White and Hispanic/Latino as well as White and some other race.

	MTurk Sample	CCES 2018	SMD
N	1468.00	21001.00	
Age (mean (SD))	37.07 (11.87)	47.03 (17.74)	0.660
Female (mean (SD))	0.56 (0.50)	0.58 (0.49)	0.032
Not female (mean (SD))	0.44 (0.50)	0.42 (0.49)	0.032
White (mean (SD))	0.69 (0.46)	0.56 (0.50)	0.281
Non-White (mean (SD))	0.31 (0.46)	0.44 (0.50)	0.281
HS or less (mean (SD))	0.09 (0.28)	0.35 (0.48)	0.674
Some college (mean (SD))	0.32 (0.47)	0.31 (0.46)	0.025
Bachelor's degree (mean (SD))	0.42 (0.49)	0.21 (0.41)	0.480
Graduate or professional degree (mean (SD))	0.17 (0.38)	0.14 (0.34)	0.097
Less than $10,000 (mean (SD))	0.04 (0.18)	0.07 (0.25)	0.143
$10,000–$19,999 (mean (SD))	0.08 (0.27)	0.09 (0.29)	0.052
$20,000–$29,999 (mean (SD))	0.11 (0.31)	0.11 (0.31)	0.001
$30,000–$39,999 (mean (SD))	0.13 (0.33)	0.11 (0.31)	0.044
$40,000–$49,999 (mean (SD))	0.11 (0.31)	0.09 (0.28)	0.080
$50,000–$59,999 (mean (SD))	0.10 (0.30)	0.08 (0.27)	0.067
$60,000–$69,999 (mean (SD))	0.09 (0.29)	0.06 (0.25)	0.092
$70,000–$79,999 (mean (SD))	0.07 (0.25)	0.07 (0.25)	0.002
$80,000–$99,999 (mean (SD))	0.09 (0.29)	0.08 (0.27)	0.046
$100,000–$149,999 (mean (SD))	0.12 (0.32)	0.10 (0.30)	0.047
$150,000 or more (mean (SD))	0.05 (0.22)	0.06 (0.24)	0.043
Prefer not to say (mean (SD))	0.03 (0.16)	0.08 (0.27)	0.242

Table S5: Comparison of Democrats in our analytic sample and in the 2018 CCES.

	MTurk Sample	CCES 2018	SMD
**N**	1070.00	17174.77	
Age (mean (SD))	40.79 (12.50)	51.40 (18.14)	0.681
Female (mean (SD))	0.58 (0.49)	0.50 (0.50)	0.164
Not female (mean (SD))	0.42 (0.49)	0.50 (0.50)	0.164
White (mean (SD))	0.87 (0.34)	0.87 (0.34)	0.011
Non-White (mean (SD))	0.13 (0.34)	0.13 (0.34)	0.011
HS or less (mean (SD))	0.11 (0.32)	0.40 (0.49)	0.686
Some college (mean (SD))	0.34 (0.48)	0.33 (0.47)	0.038
Bachelor's degree (mean (SD))	0.39 (0.49)	0.19 (0.39)	0.464
Graduate or professional degree (mean (SD))	0.15 (0.35)	0.09 (0.28)	0.187
Less than $10,000 (mean (SD))	0.03 (0.17)	0.04 (0.20)	0.055
$10,000–$19,999 (mean (SD))	0.04 (0.19)	0.07 (0.26)	0.158
$20,000–$29,999 (mean (SD))	0.09 (0.29)	0.09 (0.29)	0.001
$30,000–$39,999 (mean (SD))	0.09 (0.29)	0.10 (0.30)	0.025
$40,000–$49,999 (mean (SD))	0.10 (0.31)	0.10 (0.30)	0.023
$50,000–$59,999 (mean (SD))	0.10 (0.30)	0.09 (0.28)	0.034
$60,000–$69,999 (mean (SD))	0.10 (0.30)	0.07 (0.26)	0.103
$70,000–$79,999 (mean (SD))	0.09 (0.29)	0.08 (0.27)	0.053
$80,000–$99,999 (mean (SD))	0.14 (0.35)	0.08 (0.28)	0.182
$100,000–$149,999 (mean (SD))	0.14 (0.35)	0.11 (0.31)	0.086
$150,000 or more (mean (SD))	0.06 (0.24)	0.06 (0.24)	0.001
Prefer not to say (mean (SD))	0.01 (0.10)	0.10 (0.30)	0.410

Table S6: Comparison of Republicans in our analytic sample and in the 2018 CCES.

	Copartisan	Non-copartisan	Withheld
**Baseline**	44.47	31.20	–
**Falsification**	43.00	40.29	–
**Non-disclosure**	55.76	40.60	30.61

Table S7: Share of equitable contributions from Democratic participants, by the signaled identity of partners across experimental conditions.

	Copartisan	Non-copartisan	Withheld
**Baseline**	48.70	43.35	–
**Falsification**	40.58	41.31	–
**Non-disclosure**	44.18	39.68	39.33

Table S8: Share of equitable contributions from Republican participants, by the signaled identity of partners across experimental conditions.

	Copartisan	Non-copartisan	Withheld
**Baseline**	50.93	47.35	–
**Falsification**	45.67	44.16	–
**Non-disclosure**	62.85	37.15	32.13

Table S9: Share of equitable contributions by the identity signaled by partners, across experimental conditions; CCES weighting.

**N**	**Weighted MTurk Sample** 1269.00	**CCES 2018** 21001.00	**SMD**
Age 18–19 (mean (SD))	0.03 (0.17)	0.03 (0.17)	<0.001
Age 20–24 (mean (SD))	0.09 (0.28)	0.09 (0.28)	<0.001
Age 25–29 (mean (SD))	0.10 (0.30)	0.10 (0.30)	<0.001
Age 30–34 (mean (SD))	0.09 (0.28)	0.09 (0.28)	<0.001
Age 35–39 (mean (SD))	0.09 (0.28)	0.09 (0.28)	<0.001
Age 40–49 (mean (SD))	0.15 (0.35)	0.15 (0.35)	<0.001
Age 50–59 (mean (SD))	0.17 (0.38)	0.17 (0.38)	<0.001
Age 60 & above (mean (SD))	0.29 (0.45)	0.29 (0.45)	<0.001
Female (mean (SD))	0.58 (0.49)	0.58 (0.49)	<0.001
Not female (mean (SD))	0.42 (0.49)	0.42 (0.49)	<0.001
White (mean (SD))	0.56 (0.50)	0.56 (0.50)	<0.001
Non-White (mean (SD))	0.44 (0.50)	0.44 (0.50)	<0.001
HS or less (mean (SD))	0.35 (0.48)	0.35 (0.48)	<0.001
Some college (mean (SD))	0.31 (0.46)	0.31 (0.46)	<0.001
Bachelor's degree (mean (SD))	0.21 (0.41)	0.21 (0.41)	<0.001
Graduate or professional degree (mean (SD))	0.14 (0.34)	0.14 (0.34)	<0.001
Less than $10,000 (mean (SD))	0.07 (0.25)	0.07 (0.25)	<0.001
$10,000–$19,999 (mean (SD))	0.09 (0.29)	0.09 (0.29)	<0.001
$20,000–$29,999 (mean (SD))	0.11 (0.31)	0.11 (0.31)	<0.001
$30,000–$39,999 (mean (SD))	0.11 (0.31)	0.11 (0.31)	<0.001
$40,000–$49,999 (mean (SD))	0.09 (0.28)	0.09 (0.28)	<0.001
$50,000–$59,999 (mean (SD))	0.08 (0.27)	0.08 (0.27)	<0.001
$60,000–$69,999 (mean (SD))	0.06 (0.25)	0.06 (0.25)	<0.001
$70,000–$79,999 (mean (SD))	0.07 (0.25)	0.07 (0.25)	<0.001
$80,000–$99,999 (mean (SD))	0.08 (0.27)	0.08 (0.27)	<0.001
$100,000–$149,999 (mean (SD))	0.10 (0.30)	0.10 (0.30)	<0.001
$150000 or more (mean (SD))	0.06 (0.24)	0.06 (0.24)	<0.001
Prefer not to say (mean (SD))	0.08 (0.27)	0.08 (0.27)	<0.001

Table S10: Comparison of Democrats in our sample to those in CCES 2018 after weighting.

Range of weights	**N**	% of **N**	% of weighted **N**
$[0, 1)$	1153	78.54	21.46
$[1, 2)$	110	7.49	12.11
$[2, 3)$	45	3.07	8.85
$[3, 4)$	37	2.52	10.34
$[4, 5)$	123	8.38	47.24

Table S11: Distribution of weights among Democrats.

	Weighted MTurk Sample	CCES 2018	SMD
**N**	1269.00	17174.77	
Age 18–19 (mean (SD))	0.02 (0.15)	0.03 (0.16)	0.028
Age 20–24 (mean (SD))	0.06 (0.23)	0.06 (0.24)	0.033
Age 25–29 (mean (SD))	0.08 (0.27)	0.08 (0.27)	0.019
Age 30–34 (mean (SD))	0.05 (0.22)	0.06 (0.24)	0.058
Age 35–39 (mean (SD))	0.07 (0.26)	0.07 (0.25)	0.017
Age 40–49 (mean (SD))	0.13 (0.34)	0.14 (0.34)	0.008
Age 50–59 (mean (SD))	0.20 (0.40)	0.20 (0.40)	0.003
Age 60 & above (mean (SD))	0.39 (0.49)	0.37 (0.48)	0.036
Female (mean (SD))	0.50 (0.50)	0.50 (0.50)	0.005
Not female (mean (SD))	0.50 (0.50)	0.50 (0.50)	0.005
White (mean (SD))	0.89 (0.31)	0.87 (0.34)	0.074
Non-White (mean (SD))	0.11 (0.31)	0.13 (0.34)	0.074
HS or less (mean (SD))	0.40 (0.49)	0.40 (0.49)	0.005
Some college (mean (SD))	0.33 (0.47)	0.33 (0.47)	0.002
Bachelor's degree (mean (SD))	0.19 (0.39)	0.19 (0.39)	0.003
Graduate or professional degree (mean (SD))	0.09 (0.28)	0.09 (0.28)	0.001
Less than $10,000 (mean (SD))	0.04 (0.20)	0.04 (0.20)	0.012
$10,000–$19,999 (mean (SD))	0.08 (0.27)	0.07 (0.26)	0.018
$20,000–$29,999 (mean (SD))	0.10 (0.30)	0.09 (0.29)	0.021
$30,000–$39,999 (mean (SD))	0.11 (0.31)	0.10 (0.30)	0.022
$40,000–$49,999 (mean (SD))	0.10 (0.30)	0.10 (0.30)	0.021
$50,000–$59,999 (mean (SD))	0.09 (0.29)	0.09 (0.28)	0.020
$60,000–$69,999 (mean (SD))	0.08 (0.27)	0.07 (0.26)	0.019
$70,000–$79,999 (mean (SD))	0.08 (0.28)	0.08 (0.27)	0.020
$80,000–$99,999 (mean (SD))	0.09 (0.29)	0.08 (0.28)	0.020
$100,000–$149,999 (mean (SD))	0.12 (0.32)	0.11 (0.31)	0.024
$150000 or more (mean (SD))	0.06 (0.24)	0.06 (0.24)	0.016
Prefer not to say (mean (SD))	0.04 (0.20)	0.10 (0.30)	0.231

Table S12: Comparison of Republicans in our sample with those in 2018 CCES after weighting.

Range of weights	**N**	% of **N**	% of weighted **N**
$[0, 1)$	766	71.59	5.75
$[1, 2)$	62	5.79	6.74
$[2, 3)$	23	2.15	4.43
$[3, 4)$	24	2.24	6.69
$[4, 5)$	195	18.22	76.39

Table S13: Distribution of weights among Republicans.

	Copartisan	Non-copartisan	Withheld
**Baseline**	0.58	0.48	–
**Falsification**	0.53	0.51	–
**Non-disclosure**	0.58	0.51	0.47

Table S14: Contributions ($) by the identity signaled by partners, across experimental conditions.

	Copartisan	Non-copartisan	Withheld
**Baseline**	47.33	38.41	–
**Falsification**	43.08	43.05	–
**Non-disclosure**	53.08	40.96	34.23

Table S15: Share of equitable contributions from non-skeptical participants, by the identity signaled by partners, across experimental conditions.

N	Baseline<br>379	Non-disclosure<br>462	SMD
Female (mean (SD))	0.32 (0.47)	0.33 (0.47)	0.024
Not female (mean (SD))	0.68 (0.47)	0.67 (0.47)	0.024
White (mean (SD))	0.81 (0.39)	0.81 (0.39)	0.016
Non-White (mean (SD))	0.19 (0.39)	0.19 (0.39)	0.016
HS or less (mean (SD))	0.13 (0.34)	0.11 (0.32)	0.052
Some college (mean (SD))	0.37 (0.48)	0.39 (0.49)	0.026
Bachelor's degree (mean (SD))	0.46 (0.50)	0.48 (0.50)	0.024
Graduate or professional degree (mean (SD))	0.03 (0.17)	0.02 (0.15)	0.047

Table S16: Perceived demographic characteristics of believed Democrats in baseline and non-disclosure conditions.

N	Baseline<br>379	Falsification<br>402	SMD
Female (mean (SD))	0.32 (0.47)	0.30 (0.46)	0.045
Not female (mean (SD))	0.68 (0.47)	0.70 (0.46)	0.045
White (mean (SD))	0.81 (0.39)	0.80 (0.40)	0.022
Non-White (mean (SD))	0.19 (0.39)	0.20 (0.40)	0.022
HS or less (mean (SD))	0.13 (0.34)	0.14 (0.35)	0.022
Some college (mean (SD))	0.37 (0.48)	0.38 (0.49)	0.017
Bachelor's degree (mean (SD))	0.46 (0.50)	0.47 (0.50)	0.002
Graduate or professional degree (mean (SD))	0.03 (0.17)	0.01 (0.11)	0.116

Table S17: Perceived demographic characteristics of believed Democrats in baseline and falsification conditions.

N	Non-disclosure<br>462	Falsification<br>402	SMD
Female (mean (SD))	0.33 (0.47)	0.30 (0.46)	0.069
Not female (mean (SD))	0.67 (0.47)	0.70 (0.46)	0.069
White (mean (SD))	0.81 (0.39)	0.80 (0.40)	0.039
Non-White (mean (SD))	0.19 (0.39)	0.20 (0.40)	0.039
HS or less (mean (SD))	0.11 (0.32)	0.14 (0.35)	0.074
Some college (mean (SD))	0.39 (0.49)	0.38 (0.49)	0.009
Bachelor's degree (mean (SD))	0.48 (0.50)	0.47 (0.50)	0.022
Graduate or professional degree (mean (SD))	0.02 (0.15)	0.01 (0.11)	0.071

Table S18: Perceived demographic characteristics of believed Democrats in non-disclosure and falsification conditions.

N	Baseline<br>384	Non-disclosure<br>479	SMD
Female (mean (SD))	0.12 (0.33)	0.11 (0.31)	0.058
Not female (mean (SD))	0.88 (0.33)	0.89 (0.31)	0.058
White (mean (SD))	0.92 (0.26)	0.92 (0.27)	0.022
Non-White (mean (SD))	0.08 (0.26)	0.08 (0.27)	0.022
HS or less (mean (SD))	0.21 (0.40)	0.19 (0.39)	0.034
Some college (mean (SD))	0.35 (0.48)	0.35 (0.48)	0.016
Bachelor's degree (mean (SD))	0.42 (0.49)	0.45 (0.50)	0.061
Graduate or professional degree (mean (SD))	0.02 (0.15)	0.01 (0.12)	0.065

Table S19: Perceived demographic characteristics of believed Republicans in baseline and non-disclosure conditions.

	Baseline	Falsification	SMD
**N**	384	432	
Female (mean (SD))	0.12 (0.33)	0.12 (0.33)	<0.001
Not female (mean (SD))	0.88 (0.33)	0.88 (0.33)	<0.001
White (mean (SD))	0.92 (0.26)	0.96 (0.21)	0.133
Non-White (mean (SD))	0.08 (0.26)	0.04 (0.21)	0.133
HS or less (mean (SD))	0.21 (0.40)	0.20 (0.40)	0.005
Some college (mean (SD))	0.35 (0.48)	0.35 (0.48)	0.015
Bachelor's degree (mean (SD))	0.42 (0.49)	0.42 (0.49)	0.009
Graduate or professional degree (mean (SD))	0.02 (0.15)	0.03 (0.16)	0.027

Table S20: Perceived demographic characteristics of believed Republicans in baseline and falsification conditions.

	Non-disclosure	Falsification	SMD
**N**	479	432	
Female (mean (SD))	0.11 (0.31)	0.12 (0.33)	0.058
Not female (mean (SD))	0.89 (0.31)	0.88 (0.33)	0.058
White (mean (SD))	0.92 (0.27)	0.96 (0.21)	0.155
Non-White (mean (SD))	0.08 (0.27)	0.04 (0.21)	0.155
HS or less (mean (SD))	0.19 (0.39)	0.20 (0.40)	0.029
Some college (mean (SD))	0.35 (0.48)	0.35 (0.48)	0.001
Bachelor's degree (mean (SD))	0.45 (0.50)	0.42 (0.49)	0.051
Graduate or professional degree (mean (SD))	0.01 (0.12)	0.03 (0.16)	0.091

Table S21: Perceived demographic characteristics of believed Republicans in non-disclosure and falsification conditions.

	Democrat	Republican	Pooled
	(1)	(2)	(3)
Strength	0.076*	0.067	0.071***
	(0.035)	(0.043)	(0.027)
Constant	0.205***	0.262***	0.233***
	(0.045)	(0.055)	(0.035)
Observations	244	181	425
R^2	0.019	0.014	0.016
Note:			*p < 0.05; ** p < 0.01; *** p <0.001

Table S22: Linear probability model predicting suspicion by participant's partisanship strength (hypothesis 1).

	Democrats	Republicans	Pooled
Match	0.092	0.075	0.083
	(0.061)	(0.074)	(0.047)
Constant	0.250***	0.304***	0.277***
	(0.035)	(0.043)	(0.027)
Observations	244	181	425
R^2	0.009	0.006	0.007
Note:			*p<0.05; **p<0.01; ***p<0.001

Table S23: Linear probability model predicting suspicion by participant–partner identity match (hypothesis 2).

	Democrats	Republicans	Pooled
Distance	-0.028	-0.066	-0.047
	(0.039)	(0.047)	(0.030)
Constant	0.305^{***}	0.387^{***}	0.346^{***}
	(0.045)	(0.055)	(0.035)
Observations	244	181	425
R^2	0.002	0.011	0.006
Note:			$^{*}p<0.05$; $^{**}p<0.01$; $^{***}p<0.001$

Table S24: Linear probability model predicting suspicion by distance between participant's and partner's signaled identity (hypothesis 3).

	Copartisan	Non-copartisan	Withheld
**Baseline**	46.17	36.78	–
**Falsification**	42.35	41.08	–
**Non-disclosure**	50.93	40.57	33.57

Table S25: Share of equitable contributions by the identity signaled by partners, across experimental conditions without weights.

	Copartisan	Non-copartisan	Withheld
**Baseline**	46.14	38.02	–
**Falsification**	41.77	40.80	–
**Non-disclosure**	47.85	40.34	34.92

Table S26: Share of equitable contributions by the identity signaled by partners, across experimental conditions including all participants, i.e., participants in the baseline and non-disclosure condition who did not believe their interaction partner's party identification.

	% Fair contributions	
	**Baseline**	**Falsification**
Non-copartisan	-0.097**	-0.009
	(0.036)	(0.035)
Republican	0.058	-0.043
	(0.037)	(0.036)
18–19 years (ref.)		
20–24 years	0.223	-0.269
	(0.258)	(0.155)
25–29 years	0.150	-0.196
	(0.255)	(0.150)
30–34 years	0.038	-0.256
	(0.254)	(0.148)
35–39 years	0.202	-0.184
	(0.255)	(0.149)
40–49 years	0.164	-0.199
	(0.254)	(0.148)
50–59 years	0.195	-0.163
	(0.256)	(0.151)
60 years and above	0.322	-0.135
	(0.259)	(0.157)
Not female	-0.039	-0.078*
	(0.037)	(0.035)
Non-White	-0.024	-0.078
	(0.046)	(0.043)
HS or less (ref.)		
Some college	-0.070	-0.001
	(0.062)	(0.063)
Bachelor’s degree	-0.070	-0.051
	(0.063)	(0.062)
Graduate or professional degree	-0.116	-0.031
	(0.073)	(0.072)
Income less than $10,000 (ref.)		
$10,000–$19,999	0.016	-0.162
	-0.117	(0.126)
$20,000–$29,999	0.176	-0.109
	(0.107)	(0.116)
$30,000–$39,999	0.085	-0.040
	(0.106)	(0.114)

\$40,000–\$49,999	0.138	-0.049
	(0.11)	(0.114)
\$50,000–\$59,999	0.094	-0.106
	(0.108)	(0.114)
\$60,000–\$69,999	0.060	-0.088
	(0.108)	(0.116)
\$70,000–\$79,999	0.034	-0.017
	(0.111)	(0.120)
\$80,000–\$89,999	0.188	-0.058
	(0.121)	(0.120)
\$90,000–\$99,999	0.160	-0.098
	(0.12)	(0.124)
\$100,000–\$149,999	0.119	-0.069
	(0.107)	(0.112)
\$150,000 or more	0.219	-0.006
	(0.123)	(0.126)
Prefer not to say	0.011	-0.012
	(0.188)	(0.155)
Constant	0.258	0.785^{***}
	(0.273)	(0.179)
Observations	763	834

$^{*}p<0.05$; $^{**}p<0.01$; $^{***}p<0.001$

Table S27: Results of linear probability model predicting share of equitable contributions in the baseline and falsification conditions.

demographics

Before you begin we would like to remind you that you are going to do an activity with another person. We made a commitment to NYU's Institutional Review Board (IRB) that we would not mislead participants in any way.

On the next page you will fill out a brief questionnaire to determine whether you are eligible to complete the study.

First, we are going to ask you a few questions about yourself.

How old are you, in years?

What is your gender?

Male

Female

Something else

With which of the following groups do you identify? You may select more than one.

White

Black/African American

Hispanic/Latino(a)

Asian or Asian American

American Indian or Alaska Native

Middle Eastern or North African

Other

Where do you currently live?

Generally speaking, do you consider yourself a Republican, an Independent, a Democrat or something else?

Republican

Independent

Democrat

Something else

demographics_fail

Unfortunately, at this time we have enough people who fit your demographic profile, and you are not eligible to participate in the study. As a token of our appreciation, we would like to compensate you $0.05 for the time you have spent so far on the study. Please provide your MTurk ID in the box below, and we will follow-up about payment. Also, please return this HIT so other MTurk workers are able to take this study.

party2_questions

Where would you put yourself on this scale?

Strong Republican

Republican

Not very strong Republican

Not very strong Democrat

Democrat

Strong Democrat

DG_comp

Next, you are going to do an activity with another person.

As part of this activity, you will have the opportunity to earn up to $2.00. This is in addition to the $0.50 you will receive for completing this study. Your earnings will depend on the decisions you and the other person make in this activity. So, we encourage you to read and follow all instructions carefully.

After you finish reading the instructions, you will be asked some comprehension questions to verify that you understood them. You can only participate in the study once you have answered **all** of the questions correctly. If you do not get all questions right, you will have an opportunity to review the instructions one more time and answer the questions again. **If you fail twice you will not be able to participate in this study**.

You are going to do this activity with another person. One of you will be assigned to the role of **Player A**, and the other will be assigned to the role of **Player B**.

Player A will receive $2.00. Then, they will have to decide how much money, if any, to send to **Player B**. **Player A** can send all, some, or none of the $2.00 to **Player B**.

Both people will receive $0.50 for completing the study. As a bonus, **Player A** will receive the amount they keep from the $2.00. As a bonus, **Player B** will receive the amount **Player A** sends from the $2.00.

dg_comp_questions

Please answer these three questions carefully. You can only participate in the activity once you have answered all of them correctly. If you would like to review the instructions, please, click on the “Instructions” below.

Instructions [click here to view]

In this activity, both **Player A** and **Player B** earn a bonus. Whose decision determines the bonuses the participants earn?

Player A

Player B

Both Player A and Player B

Neither Player A nor Player B

Suppose **Player A** sends $0.50 of their $2.00 to **Player B**. What bonus does **Player A** earn?

$0.50

$1.00

$1.50

$2.00

Suppose **Player A** sends $1.00 of their $2.00 to **Player B**. What bonus does **Player B** earn?

$0.50

$1.00

$1.50

$2.00

dg_comprehension_last

You have answered one or more questions incorrectly. If you answer incorrectly again, you will not be allowed to complete the survey.

Please answer these three questions carefully. You can only participate in the activity once you have answered all of them correctly. If you would like to review the instructions, please, click on the “Instructions” below.

Instructions [click here to view]

In this activity, both **Player A** and **Player B** earn a bonus. Whose decision determines the bonuses the participants earn?

Player A

Player B

Both Player A and Player B

Neither Player A nor Player B

Suppose **Player A** sends $0.50 of their $2.00 to **Player B**. What bonus does **Player A** earn?

$0.50

$1.00

$1.50

$2.00

Suppose **Player A** sends $1.00 of their $2.00 to **Player B**. What bonus does **Player B** earn?

$0.50

$1.00

$1.50

$2.00

dg_fail

Thank you for your interest in our study. Twice, you have answered the comprehension questions incorrectly. You are no longer eligible to participate in the study. As a token of our appreciation, we would like to compensate you $0.10 for the time you have spent so far on the study. Please provide your MTurk ID in the box below, and we will follow-up about payment. Also, please return this HIT so other MTurk workers are able to take this study.

Assignment

You have been paired with another person and assigned to the role of **Player A**. The other person has been assigned to the role of **Player B**.

As **Player A**, you will receive $2.00 and you will have to decide how much money, if any, to send to **Player B**. You can send all, some, or none of the $2.00 to **Player B**.

Baseline

One more thing before you do the activity...

We have shared your answer to the following question with **Player B**:

Where would you put yourself on this scale?

- Strong Republican
- Republican
- Not very strong Republican
- Not very strong Democrat
- Democrat
- Strong Democrat

When you make your decision with the $2.00, we will also share **Player B**’s answer to this question with you.

You will be able to continue to the next page after 30 seconds have passed. While you are waiting, we encourage you to read this information carefully.

Baseline

Player B's answer to the question was **Republican.**

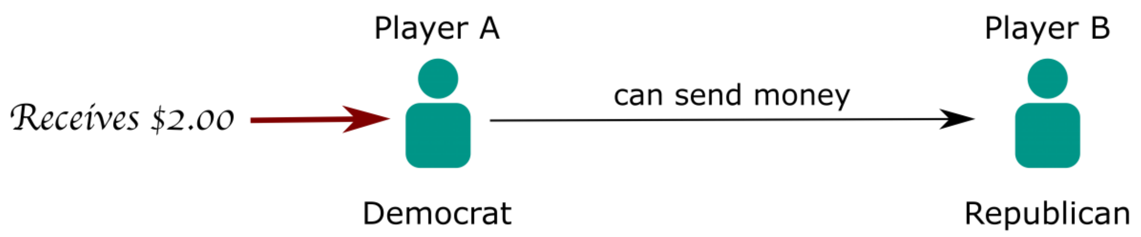

As a reminder, you have been assigned to the role of **Player A**. You have received $2.00. You can send all, some, or none of the $2.00 to **Player B**.

I am sending **Player B** $ ______

I am keeping $

ND

One more thing before you do the activity...

We have shared your answer to the following question with **Player B:**

Where would you put yourself on this scale?

- Strong Republican
- Republican
- Not very strong Republican
- Not very strong Democrat
- Democrat
- Strong Democrat

After we shared your answer to this question with **Player B**, we let them decide whether to share their answer to this question with you. When you make your decision with the $2.00, you will only receive **Player B**'s answer to this question if they decided to share it with you.

You will be able to continue to the next page after 30 seconds have passed. While you are waiting, we encourage you to read this information carefully.

Player B did not share their answer to the question. **Player B** was allowed to withhold their answer after they learned how you answered the same question.

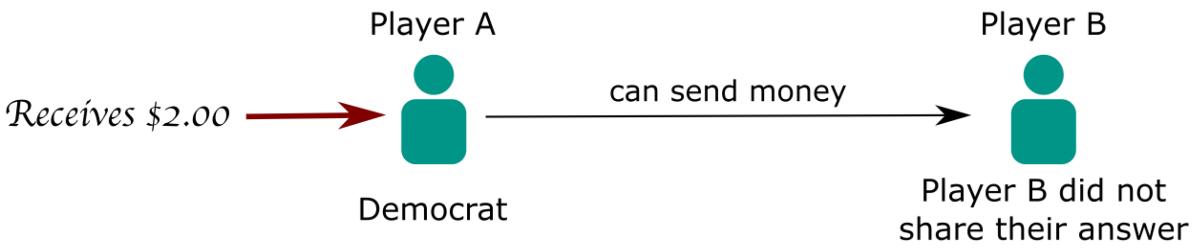

Player B's answer to the question was **Republican**. **Player B** was allowed to withhold their answer after they learned how you answered the same question.

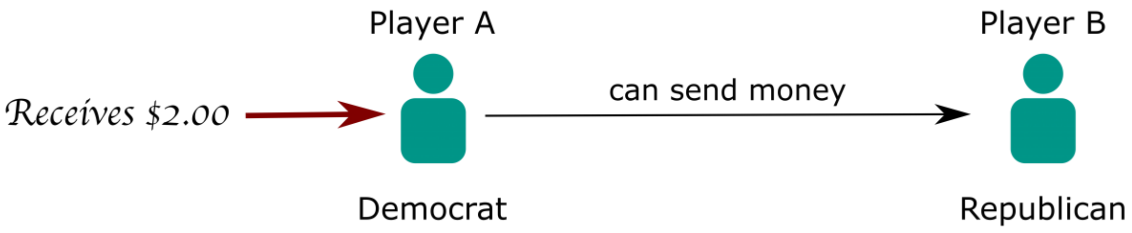

FIs

One more thing before you do the activity...

We have shared your answer to the following question with **Player B**:

Where would you put yourself on this scale?

- Strong Republican
- Republican
- Not very strong Republican
- Not very strong Democrat
- Democrat
- Strong Democrat

After we shared your answer to this question with **Player B**, we let them update their answer to this question. When you make your decision with the $2.00, you will receive only **Player B's** final answer to this question.

You will be able to continue to the next page after 30 seconds have passed. While you are waiting, we encourage you to read this information carefully.

Player B's answer to the question was **Republican**. **Player B** was allowed to change their answer after they learned how you answered the same question.

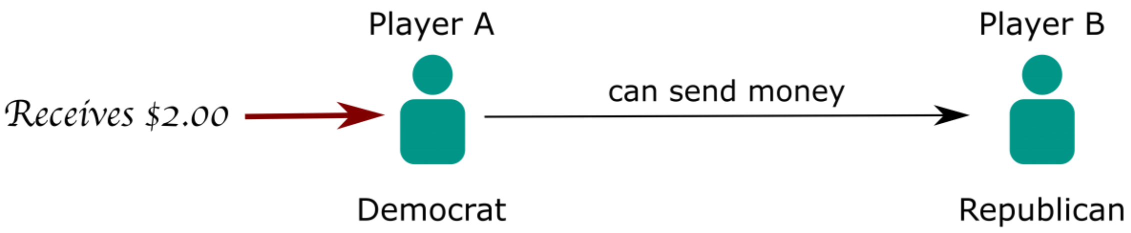

I am sending **Player B** $

I am keeping $

Follow_up

We would like to know how you made your decision about how much money to send to **Player B**. In a few sentences, please write your response in the box below.

Next, we are going to ask you a few questions about the person in the activity you just did.

What was the piece of information you saw about the other person? I was told the other person is a ...

Strong Republican

Republican

Not very strong Republican

Not very strong Democrat

Democrat

Strong Democrat

None of the above

Where do you think the other person really falls on this scale? Note: This might be the same or different from the information you saw about them.

Strong Republican

Republican

Not very strong Republican

Not very strong Democrat

Democrat

Strong Democrat

In this part of the study, we will ask you a few more questions about the other person in the activity you just did. It might be difficult to evaluate this person based on the limited information we gave you. Please do your best to answer each question, even if you may not have all the information you need. If you answer all three questions correctly, you will earn an additional bonus of$0.20.

What is the other person's gender?

Male
Female
Something else

What is the highest level of education the other person has completed?

Less than high school

High school or equivalent (e.g., GED)

Some college

2-year degree (Associate’s)

4-year degree (Bachelor’s)

Graduate or professional degree

What's the other person's race/ethnicity? You may select more than one.

White

Black/African American

Hispanic/Latino(a)

Asian or Asian American

American Indian or Alaska Native

Middle Eastern or North African

Other

Next, we are going to ask you a few questions about your views on issues facing the country.

In general, people are honest about their political views. Do you agree or disagree?

Strongly agree

Agree

Slightly agree

Slightly disagree

Disagree

Strongly disagree

Some people think that the government in Washington ought to reduce the income differences between the rich and the poor, perhaps by raising the taxes of wealthy families or by giving income assistance to the poor. Others think that the government should not concern itself with reducing this income difference between the rich and the poor.

Where would you put your views on a scale of 1 to 7, where 1 means that the government ought to reduce the income differences between rich and poor, and 7 means that the government should not concern itself with reducing income differences?

1 - The government ought to reduce income differences between rich and poor

2

3

4

5

6

7 - The government should not concern itself with reducing income differences

Next, we are going to ask you a few questions about yourself.

What is the highest level of education you have completed?

Less than high school

High school or equivalent (e.g., GED)

Some college

2-year degree (Associate’s)

4-year degree (Bachelor’s)

Graduate or professional degree

In which of these groups did your total family income, from all sources, fall last year (2018), before taxes?

Less than $10,000
$10,000 - $19,999
$20,000 - $29,999
$30,000 - $39,999
$40,000 - $49,999
$50,000 - $59,999
$60,000 - $69,999
$70,000 - $79,999
$80,000 - $89,999
$90,000 - $99,999
$100,000 - $149,999
$150,000 or more
I do not wish to report my income

For whom did you vote for president in 2016?

Hillary Clinton
Donald Trump
Someone else
I did not vote for president in 2016

Do you approve or disapprove of the way Donald Trump is handling his job as president?

Strongly approve
Somewhat approve
Somewhat disapprove
Strongly disapprove
Don't know or no opinion

What is your 5-digit ZIP code? Your answers will be kept confidential and will be used for statistical purposes only.

Thank you for taking part in the study! Before you go, we would like to get your input about the study.

What do you think was the purpose of the study? Please write your response in the box below.

Was there anything strange or surprising about the study? Please write your response in the box below.

How would you rate your overall experience completing this study?

Extremely positive

Positive

Slightly positive

Neutral

Slightly negative

Negative

Extremely negative

Powered by Qualtrics
